# Sensitizing effect of lanthanide luminescence by Mo^4+^/Ag^+^ in double perovskites: great enhancement of near-infrared emission via wide range of excitation (250–850 nm)

**DOI:** 10.1038/s41377-025-02159-4

**Published:** 2026-01-26

**Authors:** Yingsheng Wang, Peipei Dang, Zixun Zeng, Dongjie Liu, Guodong Zhang, Long Tian, Kai Li, Ping’an Ma, Yi Wei, Hongzhou Lian, Zhiyao Hou, Guogang Li, Jun Lin

**Affiliations:** 1 Key Laboratory of Rare Earth Resource Utilization, Changchun Institute of Applied Chemistry, Chinese Academy of Sciences, Changchun, Jilin China; 2 School of Applied Chemistry and Engineering, University of Science and Technology of China, Hefei, Anhui China; 3 College of Physics Science & Technology, Hebei University, Baoding, Hebei, China; 4 Faculty of Materials Science and Chemistry, China University of Geosciences, Wuhan, Hubei China; 5 Guangzhou Municipal and Guangdong Provincial Key Laboratory of Protein Modification and Degradation, School of Basic Medical Sciences, Guangzhou Medical University, Guangzhou, Guangdong China; 6 Shenzhen Research Institute, China University of Geosciences, Shenzhen, China

**Keywords:** Inorganic LEDs, Near-infrared spectroscopy

## Abstract

Lead-free halide double perovskites (LFHDPs) have gained prominence as eco-friendly optoelectronic materials due to their structural stability and flexible tunability. Lanthanide (Ln^3+^) ions have rich energy levels, which can endow LFHDP materials with emissions ranging from visible to near-infrared (NIR) region through the ion doping strategy. However, their NIR applications remain limited by narrowband emission and low photoluminescence quantum yield (PLQY) due to weak absorption cross-section. Herein, Cs_2_NaInCl_6_:Ln^3+^ were successfully synthesized, and the problem of low absorption of Ln^3+^ ions is effectively solved. Incorporating Mo^4+^/Ag^+^ ions achieves a near-unity PLQY and expands the excitation spectrum across the full visible range and a small part of NIR region (250–850 nm). Mechanism analysis revealed synergistic energy transfer pathways involving self-trapping excitons and intermediate energy states of Mo^4+^ ion, enhancing both photon absorption and PLQY. The universal applicability of this approach has been validated across Bi-based and multiple lanthanide ions (Ln: Ho, Er, Tm, Yb). These optimized materials demonstrate exceptional broadband emission characteristics suitable for multi-scenario NIR applications, including light-emitting-diodes (LEDs), night vision, imaging, anti-counterfeiting technologies. This co-doping methodology establishes a versatile framework for overcoming inherent limitations in Ln^3+^-activated materials, offering new possibilities for efficient NIR optoelectronic devices.

## Abstract (web summary)

The synthesis of Ag^+^/Mo^4^^+^/Ln^3+^ co-doped double perovskite has yielded exceptional optical properties, including broadband excitation and highly efficient NIR luminescence. These advances demonstrate promise for versatile photoelectric applications and provide critical insights into optimizing lanthanide absorption by two-step enhancement strategy.

## Graphical abstract

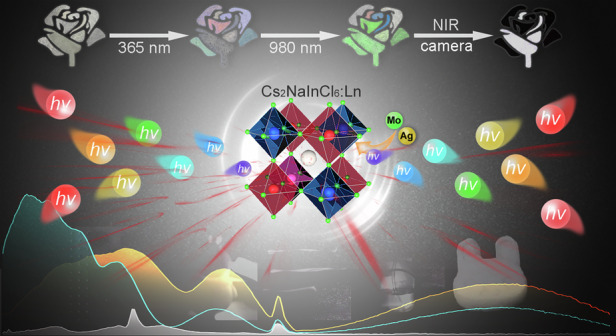

## Introduction

Lead-free metal halide perovskites (LFHPs) and their derivatives have become the research focus of a new generation of optoelectronic materials due to their unique photoelectric properties and low toxicity^1–4^. Although divalent Ge and Sn ions are close to the radius of lead ion, their inherent susceptibility to oxidation into tetravalent states induces vacancy formation and creates recombination centers, significantly reducing material stability^5–7^. Alternative non-toxic divalent and heterovalent cations often induce structural distortion due to radius mismatch and wide bandgap, resulting in losing the good properties of perovskite^8–10^. As an important branch of lead-free systems, lead-free halide double perovskites (LFHDPs, A_2_B^I^B^III^X_6_, A = Cs, Rb; B^I^ = Na, K, Ag; B^III^ = In, Bi, Ln; X = Cl, Br, I) can achieve precise regulation of band structure while maintaining the stability of perovskite structure through the orderly arrangement of B-site ion^11–13^. Due to its high chemical stability and adjustable optical properties, double perovskite is widely used in the fields of light-emitting-diodes (LEDs), photodetectors, solar cells, and X-ray detection^14–18^. However, most LFHDPs exhibit indirect bandgap, and only a few shows direct bandgap. The commercial development of these materials is hindered by low intrinsic photoluminescence (PL) intensity, which is caused by the transition of optical prohibition and challenges related to dark state self-trapping excitons (STEs)^19–21^. The doping strategy has become a critical method for fine-tuning the optical, electronic, thermal, and magnetic properties of LFHDPs, thereby overcoming their previous limitations^22,23^. For example, Na^+^-doped Cs_2_AgInCl_6_ can achieve efficient white light emission (photoluminescence quantum yields, PLQY > 86%) by regulating the symmetry of STEs to suppress non-radiative recombination^24^. The transition from indirect to direct bandgap can be achieved by adjusting the In/Bi ratio in the Cs_2_AgIn_x_Bi_1-x_Cl_6_ system (x > 0.7)^25^. In this regard, various n*s*^2^ metal ions (i.e., Sb^3+^, Te^4+^, Bi^3+^), transition metal ions with *d*–*d* transitions (i.e., Cr^3+^, Mn^2+^, Ni^2+^) and lanthanide ions with *f*–*f* transitions (i.e., Ho^3+^, Er^3+^, Tm^3+^, Yb^3+^) have been established as effective dopants for various LFHDPs^26–29^.

With the wide applications of near-infrared (NIR) light sources in biological imaging, night vision, non-invasive detection, and other fields^30–33^, the emerging lanthanide ions (Ln^3+^) doped LFHDP NIR emitters stand out among many materials due to their superior cost-effectiveness, high efficiency, and long life^34,35^. However, there are still significant bottlenecks in the application of these materials for NIR luminescence. First, the weak exciton binding energy in LFHDPs promotes non-radiative recombination pathways, resulting in unsatisfactory PLQY (<10%)^36^. Second, the intrinsic *f*–*f* transition of Ln^3+^ in Ln^3+^-doped materials restricts their emission spectra to narrowband (the full width at half maximum, FWHM < 100 nm), which is difficult to meet the needs of wide-spectrum NIR light sources for applications^37–39^. Therefore, the development of LFHDP materials with high PLQY and broadband NIR emission has become a key issue to be solved in these fields. Transition metal ions doping has emerged as a promising strategy to address these limitations^23,28^. By introducing intermediate energy states through *d*-orbital hybridization, this approach enables effective bandgap engineering and enhances photon absorption cross-sections^40^. Therefore, the introduction of transition metal ions can effectively improve the absorption efficiency of Ln^3+^ ions and create more application prospects for Ln^3+^-doped LFHDPs. For example, thanks to the sensitization of Cr^3+^ ion, the excitation range of Cs_2_AgInCl_6_:Er^3+^ has been greatly improved. After annealing, the PLQY value of the Cr^3+^/Er^3+^ co-doped sample can reach as high as 57.5%^41^.

In our previous work^42^, transition metal Fe^3+^ is used to sensitize Ln^3+^ (Ln: Dy, Tm, Er), enabling satisfactory NIR-II imaging performance with blue light (450 nm) excitation. Ln^3+^ can be effectively excited by irradiation ranging from 250 to 550 nm with maximum at 450 nm thanks to Fe^3+^ doping. However, there are several limitations in this system: while Fe^3+^ successfully sensitizes Ln^3+^, it lacks its intrinsic luminescence properties. Furthermore, the single *f*–*f* transitions of Ln^3+^ are insufficient for multi-scenario NIR applications with a internal quantum efficiency (IQE) of 20%. It is well-known that expanding the material’s absorption range could significantly enhance the application potential of Ln^3+^-doped luminescent materials. Recently, Mo^4+^ ion has gained much attention due to its excellent light absorption and NIR luminescence properties^43–45^. Its *d*^2^ electronic configuration might be a good choice for sensitizing Ln^3+^. However, its potential for improving Ln^3+^ emission remains unexplored. Motivated by this gap, we investigate Mo^4+^/Ln^3+^ co-doping to assess the feasibility of sensitization strategy in double peroskites. In this study, we synthesized Cs_2_NaMCl_6_:Ln^3+^ (M: In, Bi; Ln: Ho, Er, Tm, Yb) double perovskite using the solvothermal method. Impressively, the luminescence efficiency can be enhanced twice by separately introducing Mo^4+^ and Ag^+^ ions, even reaching the near-unity PLQY. It is quite remarkable that after the two doping processes, the excitation range of Ln^3+^ is expanded to cover the entire visible region and a small part of the NIR region (250–850 nm). The luminescence intensities of Ln^3+^ ions have also increased by tens or even thousands of times. Through temperature-dependent PL spectra and first-principles calculations, we can attribute this phenomenon to the generation of multiple energy transfer pathways including STEs and intermediate energy states of Mo^4+^ ion, enhancing both photon absorption and PLQY. Moreover, this phenomenon is applicable to both Bi-based and In-based double perovskite systems. Finally, we conducted extensive explorations into the applications of these properties and discovered promising potential for NIR LEDs, night vision, imaging, and anti-counterfeiting technologies. This innovative methodology not only resolves long-standing limitations in Ln^3+^-activated materials, but also unlocks promising potential for multi-scenario NIR optoelectronic applications.

## Results

**Fig. 1:**
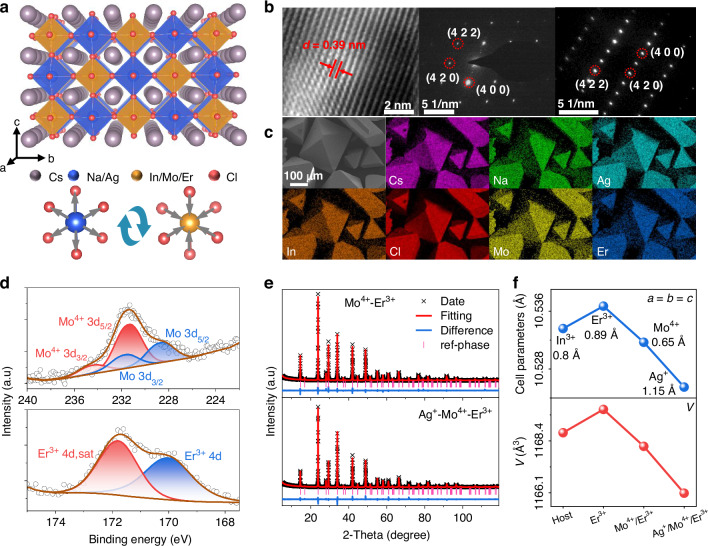
Structure and morphology characterizations of doped Cs_2_NaInCl_6_. **a** Crystal structure of NAIME double perovskite and the structural evolution after dopant incorporation. **b** The SAED and HRTEM images of NIME (left) and NAIME (right) SCs. **c** SEM and EDS elemental mapping images of NAIME SCs. **d** High-resolution XPS patterns of Mo^4+^ 3 *d* and Er^3+^ 4 *d* in NAIME. **e** XRD Rietveld refinement of the as-prepared NIME and NAIME samples. **f** Systematic variation of unit cell parameters and volume for the host, Er^3+^-, Mo^4+^/Er^3+^- and Ag^+^/Mo^4+^/Er^3+^-doped samples

Representatively, a series of Er^3+^-, Mo^4+^/Er^3+^- and Ag^+^/Mo^4+^/Er^3+^-doped Cs_2_NaInCl_6_ (named as NIE, NIME and NAIME, respectively) samples were synthesized via solvothermal method using metal halide, MoO_3_ and concentrated HCl as precursors. This approach can obtain double perovskite single crystals (SCs) with the size from hundreds of micrometers to several millimeters (Fig. S1). The host follows the characteristic cryolite structure with Fm-3m space group, comprising alternating [NaCl_6_]^5-^ and [InCl_6_]^3-^ octahedron interspersed by Cs^+^ ion. Mo^4+^ and Er^3+^ ions were designed to substitute In^3+^ sites, forming [MoCl_6_]^2-^ and [ErCl_6_]^3-^ octahedra within the host lattice (Fig. 1a). Powder X-ray diffraction (PXRD) patterns for all compositions are consistent with the Cs_2_NaBiCl_6_ standard card (PDF#70-1420), confirming the formation of phase-pure solid solutions (Fig. S2). Selected-area electron diffraction (SAED) analysis reveals well-defined crystalline diffraction spots, verifying the single-crystalline nature of the as-prepared samples (Fig. 1b). Through systematic indexing of the diffraction pattern, three dominant crystallographic planes (4 0 0), (4 2 2), and (4 2 0) are unambiguously identified. High-resolution transmission electron microscopy (HRTEM) further reveals highly ordered atomic arrangements, evidenced by lattice fringes with interplanar spacings of 0.39 nm, corresponding to the (2 2 0) plane of the NIME phase. Scanning electron microscope (SEM) images display regular octahedral morphologies, while energy dispersive spectrometer (EDS) mapping images confirm homogeneous distribution of Ag^+^, Mo^4+^ and Er^3+^ elements among the host microcrystals (Fig. 1c). X-ray photoelectron spectroscopy (XPS) analysis validates the oxidation states of the doping ions (Figs. S3–5). Characteristic peaks at 231.3 eV and 234.4 eV correspond to Mo^4+^ 3*d*_5/2_ and 3*d*_3/2_ states, respectively, while the 170.0 eV peak corresponds Er^3+^ 4 *d* configuration (Fig. 1d)^46,47^. It is worth noting that, although MoO_3_ was used as the precursor, the photoluminescence (PL) spectrum of Mo ion was the same as that obtained using MoO_2_ (Fig. S6). Therefore, it can be concluded that the Mo ion entering the lattice is +4 valence. By using SC XRD analysis, the detailed parameter information of host, Mo^4+^-, and Mo^4+^/Er^3+^-doped samples are provided in Table S1. Subsequently, the Rietveld refinements of PXRD data were conducted using the obtained CIF files, yielding the results presented in Fig. S7 and Fig. 1e. The crystallographic parameters obtained from XRD Rietveld refinements are shown in Table S2. The unit cell parameters evolve systematically with dopant introduction, it can be seen that sequential incorporation of Er^3+^ (*r* = 0.89 Å), Mo^4+^ (*r* = 0.65 Å) and Ag^+^ (*r* = 1.15 Å) ions initially increases the lattice parameters before a subsequent contraction (Fig. 1f). This behaviors of Er^3+^ and Mo^4+^ ions align with the size-dependent lattice evolution of ion radius, while Ag^+^ ion cause further unit cell shrinkage. Bond length analysis indicates preferential expansion of [NaCl_6_]^5-^ octahedra and compression of [InCl_6_]^3-^ units, resulting in the reduced unit cell volumes (Table S3). A comprehensive analysis of lattice contraction will be presented in the following section. Inductively coupled plasma-optical emission spectrometry (ICP-OES) confirms precise correspondence between nominal and actual doping concentration (Table S4). It shows that even if the feeding ratio is large, the actual amount of incorporation is very small due to solubility and isovalent substitution, etc (Fig. S8).

**Fig. 2:**
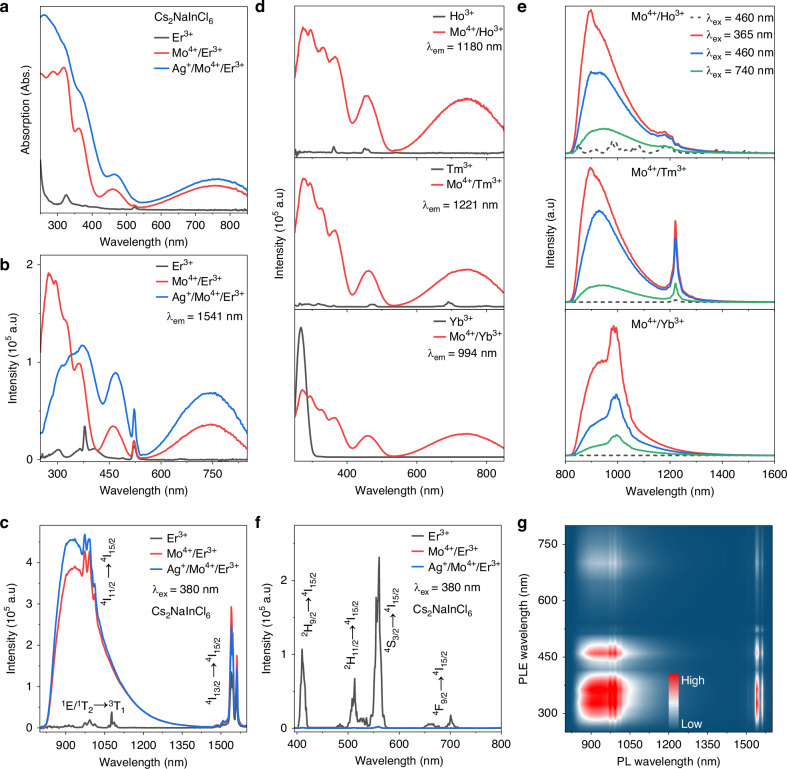
Photoluminescence properties of Mo^4+^/Ag^+^ doped Cs_2_NaInCl_6_:Ln^3+^. **a** Absorption spectra, **b** PLE spectra and **c** NIR PL spectra of NIE, NIME, and NAIME at RT. The **d** PLE and **e** NIR PL spectra of Cs_2_NaInCl_6_:Mo^4+^/Ln^3+^ (Ln: Ho, Tm, Yb) under different excitation (the excitation conditions of Cs_2_NaInCl_6_:Mo^4+^/Ln^3+^ are consistent, and the gray dotted lines are the PL spectra of Ln^3+^ singly doped materials) and emission wavelengths. **f** The visible PL spectra of NIE, NIME, and NAIME at RT (the red and blue lines basically overlap together with very weak luminescence signals). **g** Excitation wavelength-dependent PL spectra of NIME measured at RT (color scales label correspond to relative luminescence intensity)

As shown in Fig. 2a, the NIE exhibits only two weak absorption bands in the visible range peaking at 325 and 520 nm, corresponding to bandgap absorption and the characteristic transition ^4^I_15/2_ → ^2^H_11/2_ of Er^3+^ ion, respectively^48^. In contrast, the NIME demonstrates significantly enhanced broadband absorption spanning 250–850 nm, primarily attributed to Mo^4+^-related electronic transitions^43^. Monitored by the characteristic emission of Er^3+^ at 1541 nm (^4^I_13/2_ → ^4^I_15/2_), the obtained photoluminescence excitation (PLE) spectrum of NIME consists of four distinct excitation bands with maxima at 300, 365, 460 and 740 nm, respectively (Fig. 2b). The 300 nm band is assigned to Mo-Cl charge transfer (CT) and host lattice band-edge transitions, while the remaining bands (365, 460 and 740 nm) correspond to the *d*–*d* transitions of Mo^4+^ ion: ^3^T_1_(^3^F) → ^3^T_1_(^3^P), ^3^T_1_(^3^F) → ^3^T_2_(^3^F) and ^3^T_1_(^3^F) → ^1^E/^1^T_2_(^1^D), respectively^43,49^. Meanwhile, the PLE measured by monitoring the emission of Mo^4+^ (930 nm) and Er^3+^ (1541 nm) ions are basically consistent and there is spectral overlap between Mo^4+^ ion emission and Er^3+^ ion excitation spectra (Fig. S9). These clearly indicate that an energy transfer (ET) occurs from Mo^4+^ to Er^3+^ in the host lattice. Notably, the incorporation of Ag^+^ ion further enhances the light absorption of Er^3+^ ion. This enhancement may be attributed to the formation of self-trapped excitons (STEs) induced by Ag^+^ doping^24^. The existence of STEs was confirmed by the visible PL spectra and lifetime decay curves of NAIME and NAI. The broad-spectrum profile of NAIME is similar to that of NAI and the lifetime is in microseconds, which is consistent with the characteristics of STEs emission in Tang’s work (Fig. S10)^24^. The PL spectra of Mo^4+^/Er^3+^ co-doped systems show additional near-infrared (NIR) emission beyond the characteristic emission (^4^I_13/2_ → ^4^I_15/2_) of Er^3+^. Fig. 2c displays a broad non-Gaussian NIR emission band spanning 750–1400 nm (peak at 930 nm, FWHM ≈ 180 nm), arising from the radiative transition ^1^E/^1^T_2_(^1^D) → ^3^T_1_(^3^F) of Mo^4+^ ion^43^. The enhancement effect exhibits a considerable universality, as evidenced by similar observations in both Ln^3+^-doped (Ln: Ho, Er, Tm, Yb) and Bi-based double perovskites (Figs. 2d, e, S11). Similar phenomena also exist in other Ln-based samples such as Gd and Lu, but not obvious (Fig. S12). It can be seen that the Mo^4+^ ion significantly enhance the PL emission of Ln^3+^ ions. Under the excitation of 460 nm light, the luminescence intensities of Ho^3+^, Er^3+^, Tm^3+^ and Yb^3+^ are amplified by 18-, 74-, 36- and 1774-fold in In-based system, respectively (Fig. S13). Correspondingly, in Bi-based systems, their luminescence intensities are enhanced by 6-, 134-, 31- and 3142-fold, respectively. In addition, photoluminescence quantum yield (PLQY) has also been improved. Taking Er^3+^ ion as a representative case, the internal quantum efficiency (IQE), absorption efficiency (Abs) and external quantum efficiency (EQE) of NIE, NIME and NAIME under the excitation of 365, 380, 460, and 740 nm light are characterized (Fig. S14). After two-step enhancement strategy, the IQE even reaches up to 100%, outperforming most reported Ln^3+^-doped LFHPs (Table S5). Similar to our previous work for Fe^3+^/Er^3+^ co-doping system, the introduction of Mo^4+^ ion suppresses the visible region luminescence of Er^3+^ ion but dramatically enhances NIR emissions (Fig. 2c, f)^42^. It is assumed that the incorporation of Mo^4+^ alters the population distribution of Er^3+^ energy levels, preferentially stabilizing electrons in lower-energy states. The distinction of this work lies in its dual achievement: not only does it enhance Mo^4+^-related absorption, but it also addresses the previous deficiency in NIR-I spectral coverage. The excitation wavelength-dependent PL spectra of NIME measured at room temperature (RT) show the luminescence intensities of both emission bands change uniformly with varying excitation wavelength, suggesting the presence of only two distinct emission centers and broad excitation range (Fig. 2g). Furthermore, the PLE spectra recorded at different PL emission wavelengths exhibit nearly identical spectral features, further confirming that Er^3+^ luminescence may derive energy from the absorption of Mo^4+^ ion through ET process from Mo^4+^ to Er^3+^ ion (Fig. S15).

**Fig. 3:**
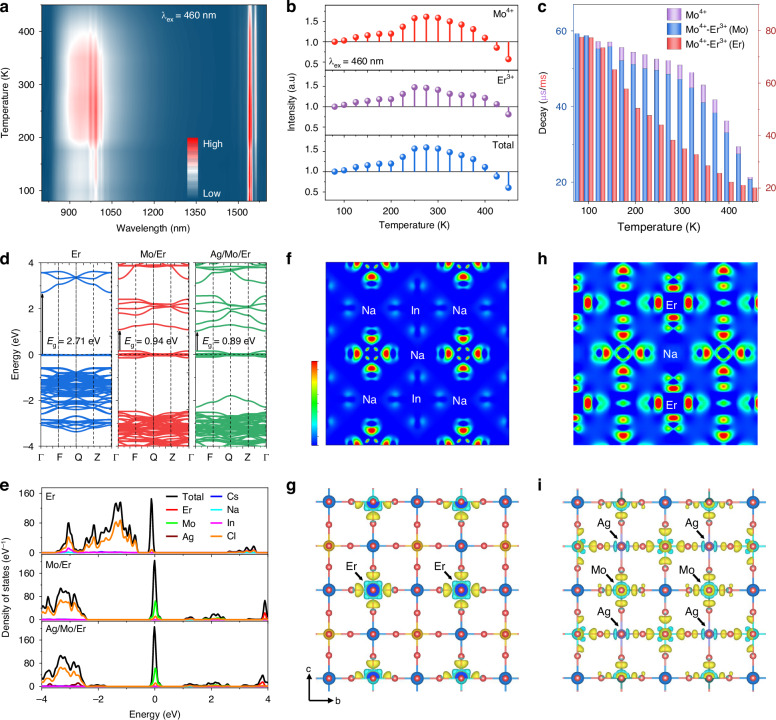
Temperature-dependent PL properties and DFT calculation. **a** The contour plot of temperature-dependent PL spectra of NIME. **b** Temperature-dependent PL intensity variations of NIME (intensity refers to the integral intensity). **c** Comparative temperature-dependent lifetimes for NIM and NIME systems. **d** The electronic band structure comparison among NIE, NIME, and NAIME. **e** The pDOS patterns for NIE, NIME, and NAIME. The charge density distribution for **f** NIE and **h** NAIME. The differential charge distribution for **g** NIE and **i** NAIME. The blue, purple, yellow, red, dark green, and turquoise balls represent Na, Ag, In, Cl, Mo, and Er atoms, respectively

To obtain more perspectives on luminescent properties, temperature-dependent PL spectra and lifetimes were explored. Figure 3a presents the PL spectral contour map of NIME under 460 nm excitation measured from 80 and 450 K. Additional spectral contour plots of NIM and those of NIME monitored at other wavelengths are shown in Figs. S16 and S17, respectively. It can be observed that the PL intensity remains essentially stable from 80 to 200 K. In contrast, within the 200–400 K regime, a distinct two-stage behavior emerges: initial luminescence enhancement followed by progressive thermal attenuation (Figs. 3b, S18). At lower temperatures (<200 K), the observed intensity reduction is attributed to the enhanced electron–phonon coupling effect that promotes non-radiative recombination pathways, as evidenced by shortened luminescence lifetimes (Fig. 3c). Conversely, the anomalous intensity increase at elevated temperatures (>200 K) stems from thermally activated lattice softening, which facilitates vibration coupled *d*–*d* transitions and liberates trapped charge carriers^50^. To quantitatively assess electron–phonon coupling effect, the Huang-Rhys factors (*S*) of NIME and NAIME are determined by analyzing the temperature-dependent evolution of PL FWHM (Fig. S19). The detailed calculation methods are shown in the experimental section of Supporting Information (SI). The *S* values of NIME and NAIME obtained by fitting are 3.3 and 3.8, respectively, indicating weak electron–phonon coupling. This also verifies that the NIR-I emission belongs to *d*–*d* transitions rather than STE with a usually *S* value greater than 30^51^. In addition, the increased *S* factor in Ag^+^ incorporated samples exhibits that the lattice distortion is more pronounced. The comparable temperature-dependent intensity profiles between Er^3+^ and Mo^4+^ ions suggest that the ET process from Mo^4+^ to Ln^3+^ contributes to the enhanced thermal stability of the Ln^3+^ ions. The lifetime curves of Er^3+^ ion in NIME show an upward trend before decaying, revealing temperature-dependent ET dynamics (Fig. S20). The lifetime variation of Mo^4+^ ion in NIM and NIME systems implies non-monotonic ET efficiency changes as the temperature increases (Fig. 3c).

In addition, the electronic properties of NIE, NIME, and NAIME double perovskites were systematically investigated using first-principles density functional theory (DFT) calculations. To support subsequent mechanism analysis, structural optimization identifies that the most stable Mo^4+^ substitution configuration is In^3+^ site, and lattice parameters are provided in Table S6. Among the four possible substitution configurations, the In^3+^ site exhibits the highest binding energy for Mo^4+^ substitution, confirming its thermodynamic stability (Fig. S21). Unit cell parameter analysis enables reconstruction of elemental octahedral geometries (Fig. S22), showing consistency with the structural features presented in Fig. 1f. Furthermore, atom-projected partial density of states (pDOS) and electronic band structures analysis reveal critical insights into the electronic behavior. It should be noted that the DOS is intentionally projected onto only the valence electron orbitals to elucidate their distinct electronic contributions, as this is the primary focus of our study. Therefore, the sum of the pDOS is not expected to be equal to the full TDOS. Band structure calculations confirms that NIE exhibits a direct bandgap semiconductor (*E*_*g*_ = 2.71 eV), with both valence band maximum (VBM) and conduction band minimum (CBM) located at the Γ point (Fig. 3d). The VBM primarily originates from Cl 3*p* orbitals, while the CBM arises from hybridization between In 5 *s* and Cl 3*p* orbitals (Fig. 3e). Notably, Mo^4+^ substitution induces the formation of mid-level states through hybridization between Mo 4 *d* and Cl 3*p* orbitals, while strong polarization effect from these interactions conferred half-metallic characteristic to the alloy systems. The half-metallic behavior manifests as metallic properties with zero bandgap in the majority-spin channel, while the minority-spin channel retains semiconductor or insulator characteristics^50^. The half-metallic character of the alloy system is explicitly demonstrated through electronic band structure and pDOS analysis, which reveals pronounced orbital hybridization and finite electronic states at the Fermi level. Meantime, the substitution of Ag^+^ is not obvious in pDOS. The charge density distribution and differential charge distribution demonstrate nearly complete ionization of Na^+^ ion in NIE, with electron density highly localized within [ErCl_6_]^3-^ octahedra. The low charge density near Na^+^ and In^3+^ sites indicates the presence of dark STEs in the host lattice (Fig. 3f, g)^24^. Mo^4+^ substitution enhances covalent interactions in Mo-Cl bonds, as evidenced by differential charge density distributions (Fig. S23). It is worth nothing that the Fermi level proximity to CBM in NIME facilitates enhanced electron transition probabilities, significantly improving optical absorption. Furthermore, the introduction of Ag^+^ ion alters behavior of the system through the following two aspects. On the one hand, Ag^+^ substitution breaks the forbidden transitions and alters parity of the wavefunction of STEs, transforming the dark STEs into a bright STEs. On the other hand, compared with Na^+^ ion, the electron covalency of Ag^+^ ion is significantly increased, and the Ag-Cl interaction is also increased. Hole trapping at Ag sites produces a 4*d*^9^ electronic configuration, inducing Jahn-Teller (J-T) distortions (Fig. 3h, i). The synergistic effects of Mo^4+^ substitution (inducing half-metallicity and bandgap modulation) and Ag^+^ incorporation (enhancing electron transition probabilities via STE modification) collectively drive the observed PL property enhancements.

**Fig. 4:**
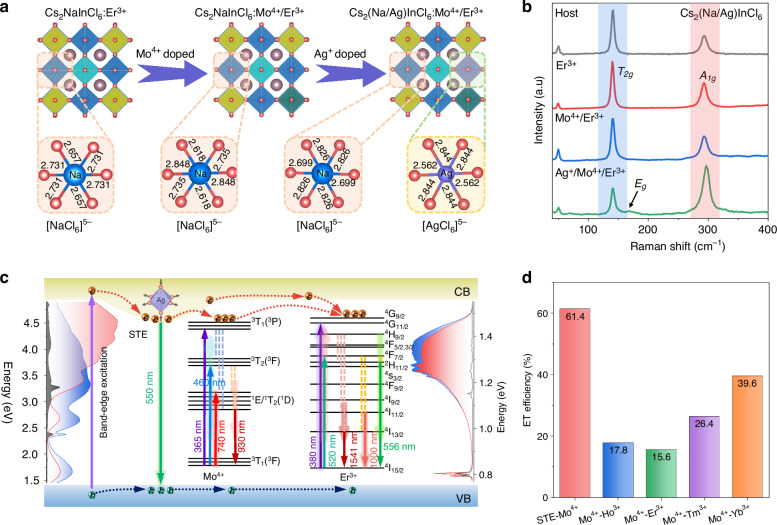
Mechanism explanation and energy transfer efficiency. **a** Schematic diagram of sublattice distortion in Cs_2_NaInCl_6_:Er^3+^ caused by Mo^4+^ and Ag^+^ ions doping. Yellow, blue, turquoise, dark green, and purple octahedra represent [InCl_6_]^3-^, [NaCl_6_]^5-^, [ErCl_6_]^3-^, [MoCl_6_]^2-^, and [AgCl_6_]^5-^, respectively. **b** Comparative Raman spectra of pristine and doped samples. **c** Mechanism diagram of NIR emission enhancement. **d** The calculated ET efficiencies of different pathways

The lattice deformation behavior in double perovskite samples after alloying was systematically investigated, as illustrated in Fig. 4a. The introduction of Mo^4+^ and Ag^+^ ions induces distortion in [NaCl_6_]^5-^ and [InCl_6_]^3-^ octahedra. Bond length analysis reveals that both [NaCl_6_]^5-^ and [AgCl_6_]^5-^ octahedra exhibit equatorial plane expansion and axial contraction, whereas the [InCl_6_]^3-^ octahedron exhibits the opposite trend-compression in the equatorial plane and expansion along the vertical axis, reducing local octahedral symmetry (Fig. S22). This also explains the observed lattice contraction upon Ag^+^ introduction. Raman spectroscopic analysis proves two distinct vibrational modes (Fig. 4b). The low-frequency band of the *T*_*2g*_ mode centered at 123 cm^−1^ is attributed to the bending vibration of [InCl_6_]^3-^ and [NaCl_6_]^5-^ octahedra, while the *A*_*1g*_ mode centered at 290 cm^−1^ is related to their symmetric stretching vibration^52^. Notably, the previously obscured *E*_*g*_ mode associated with asymmetric stretching of [InCl_6_]^3-^ octahedra emerges upon Ag^+^ doping, accompanied by the enhancement of *A*_*1g*_ peak and the attenuation of *T*_*2g*_ peak. These spectral modifications are attributed to J-T distortion, which enhances electron–phonon coupling and relaxes selection rules^53^. Based on the above discussion, we propose a second-order enhancement mechanism for Ln^3+^ ions in double perovskites. As shown in Fig. 4c and Fig. S24, the introduction of Mo^4+^ ion introduces *d*–*d* orbital transitions and half-metallic characteristics via unique hybridization between Mo 4 *d* and Cl 3*p* orbitals. Meanwhile, the spin-allowed transitions of Mo^4+^ ion greatly improve the absorption efficiency of Ln^3+^ ions through ET process. The Tanabe-Sugano (T-S) diagram can intuitively show the transition path of the *d*^2^ electronic configuration for Mo^4+^ ion (Fig. S25). Although the lowest energy transition ^3^T_1_ → ^1^E/^1^T_2_ is a both Laporte- and spin-forbidden transition formally, the transition becomes partially allowed due to strong spin-orbit coupling and [MoCl_6_]^2-^ octahedral distortion^43^. Furthermore, the introduction of Ag^+^ ion with *d*^9^ electronic configuration leads to additional J-T distortion, converting the original dark STEs into bright STEs and improving the absorption of material. The time-resolved PL (TRPL) spectra confirm a rapid decay of Mo^4+^ ion emission contrasted with a prolonged decay of Ln^3+^ ion emissions (Fig. S26). These results strongly implied that Mo^4+^ ion as both sensitizer and activator, enables the effective Mo^4+^-Ln^3+^ ET. This strategy demonstrates versatility across multiple Ln^3+^ co-doped systems and maintains excellent thermal stability (Fig. S27). Finally, the ET efficiencies (*η*_ET_) can be calculated for STEs-Mo^4+^ (61.4%), Mo^4+^-Ho^3+^ (17.8%), Mo^4+^-Er^3+^ (15.6%), Mo^4+^-Tm^3+^ (26.4%) and Mo^4+^-Yb^3+^ (39.6%) in Cs_2_NaInCl_6_ system, respectively (Fig. S28–29)^54^. The luminescence lifetime of Mo^4+^ ion and the calculated ET efficiencies at various concentrations of acceptors are presented in Table S7–11. Moreover, the ET from Mo^4+^ to Ln^3+^ at various temperatures were also characterized. The observed trend closely aligns with the temperature-dependent variation in luminescence intensity, suggesting that the ET process has excellent thermal stability (Fig. S30).

**Fig. 5:**
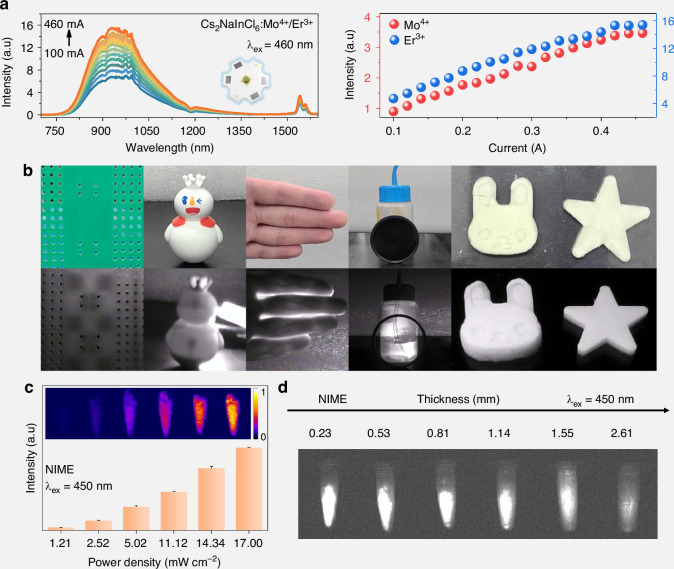
Electroluminescence properties and NIR applications. **a** Driven current-dependent EL spectra and EL intensity of the as-fabricated NIR pc-LED with 460 nm chip. **b** The NIR imaging capabilities of the as-fabricated pc-LED. (The upper panel displays visible-light images captured by visible camera in natural light, while the lower panel was taken with NIR camera under pc-LED illumination). **c** The histogram of NIR luminescence intensity excited with different power densities. The insets are NIR luminescence images of NIME excited with different power densities. **d** The NIR luminescence images of NIME through seal films of different thicknesses under the excitation of 450 nm

In order to explore the application potential of the studied materials, NIME was integrated with commercial LED chips (λ_em_ = 365 nm and 460 nm) to construct NIR phosphor-converted light-emitting-diodes (pc-LEDs). Figure 5a shows the current-dependent electroluminescence (EL) spectra of the 460 nm chip-based pc-LED. The EL intensity of NIR exhibits progressive enhancement as the driving current increases from 100 to 400 mA without obvious saturation phenomenon. Different from the PL spectrum, the relative intensity of Mo^4+^ and Er^3+^ ions changed, which may be caused by the difference of detectors and chip. A similar monotonic trend is observed for the 365 nm chip-based device, confirming robust operational stability (Fig. S31). Thermogravimetric analysis reveals negligible mass loss for both pristine and doped samples up to 600 °C, suggesting excellent thermal stability (Fig. S32). Moreover, the samples retained >95% of initial PL intensity after three months ambient storage, indicating remarkable environmental stability (Fig. S33). Subsequently, the pc-LED was carefully evaluated for night vision and imaging applications. As shown in Fig. 5b, NIR light from the device penetrates non-transparent circuit board, enabling non-destructive inspection of internal integrated circuits. Similarly, the iron block inside the tumbler is clearly seen. In addition, the remarkable tissue-penetrating capability of NIR light enables precise visualization of subcutaneous vascular networks in human fingers through the optical imaging technique. Ethanol meniscus visibility, obstructed by a 750 nm filter under ambient light, is restored via pc-LED illumination. To validate NIR imaging versatility, we embedded NIME powder into polydimethylsiloxane (PDMS) to prepare NIME-PDMS composite patterns. The patterns can exhibit controllable excitation-responsive luminescence under 460 nm irradiation. The blue-excitable NIR emission of the material makes it highly suitable for NIR imaging applications. As shown in Fig. 5c and Fig. S34, the NIR luminescence intensities of NIMH, NIME, NIMT, and NIMY were systematically measured under varying excitation power densities. The results reveal a clear power-dependent enhancement in NIR emission, demonstrating the promising potential for high-performance NIR imaging. To further assess the tissue penetration capability of the NIR luminescence, we conducted depth-dependent measurements using sealing films of varying thicknesses^55^. Although the luminescence intensity decreased with increasing thickness, the NIR imaging exhibited a strong signal-to-noise ratio and maintained detectable emission signals at penetration depths exceeding 2.6 mm (Fig. 5d and Fig. S35).

**Fig. 6:**
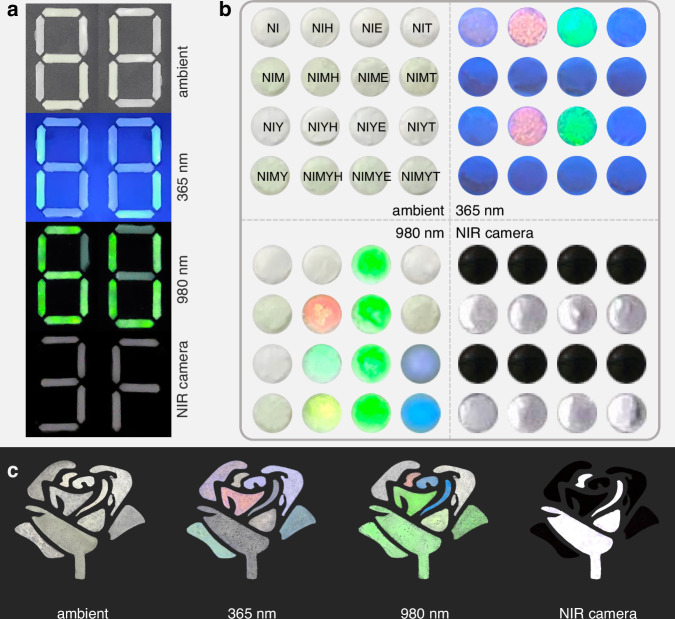
Information encryption and anti-counterfeiting applications. **a** Multi-mode information encryption application display. **b** The multi-mode luminescence of each sample studied. **c** The anti-counterfeiting pattern made by combining several different samples

The up-conversion (UC) luminescence of Er^3+^ ion is well known, which endows the material with enhanced functionality, thereby broadening its potential applications (Fig. S36)^48^. We developed a multi-mode information encryption platform based on NIE/NIM/NIME-PDMS composites. Leveraging the multi-mode luminescence of Er^3+^ ion, a reconfigurable seven-segment display was designed in Fig. 6a. The composites displayed: (i) null emission (“88”) under ambient light; (ii) visible “3 F” under 460 nm excitation by NIR camera; (iii) visible-light “1 J” under 365 nm UV light; and (iv) UC luminescence “6U” under 980 nm laser irradiation. Additionally, a series of Mo^4+^/Yb^3+^/Ln^3+^ co-doped Cs_2_NaInCl_6_ (Ln = Ho, Er, Tm) were synthesized, and their multi-modal luminescence properties were systematically characterized (Fig. S37). The corresponding emission colors are presented in Fig. 6b. To further validate the practical utility of these materials, an anti-counterfeiting pattern was fabricated using these synthesized samples, as illustrated in Fig. 6c. By 365 nm and 980 nm excitation and detecting with NIR camera, the “flower” pattern demonstrates distinct luminescence responses. In general, these findings highlight the potential of the as-studied materials in applications such as night vision, NIR imaging and information encryption.

## Discussion

In summary, we successfully synthesized the Cs_2_NaMCl_6_:Ln^3+^ (M: In, Bi; Ln: Ho, Er, Tm, Yb) double perovskite via a solvothermal method and developed an innovative strategy to significantly enhance its luminescence performance. By sequentially introducing Mo^4+^ and Ag^+^ ions, a two-stage amplification of photoluminescence quantum yield (PLQY) is achieved, ultimately reaching near-unity values in Er^3+^-doped materials. Remarkably, this synergistic doping protocol expanded the excitation spectrum across the entire visible region and a small part of the near-infrared (NIR) region (250–850 nm), enabling broadband photon absorption. It is noteworthy that the luminescence intensities of Ln^3+^ (Ho^3+^, Er^3+^, Tm^3+^ and Yb^3+^) ions are enhanced by 18-, 74-, 36- and 1774-fold after the introduction of Mo^4+^ ion in In-based system, respectively. Temperature-dependent PL measurements demonstrate the exceptional thermal stability, with luminescence intensity maintaining unchanged and even exhibiting slight thermal enhancement. Furthermore, the first-principles calculations reveal that the enhanced performance arises from the establishment of multiple energy transfer (ET) pathways. Specifically, as a dual-functional ET medium, Mo^4+^ ion act as both an activator to absorb excitation energy and a sensitizer to efficiently transfer the absorbed energy to Ln^3+^ ions, increasing the absorption and luminescence intensity of Ln^3+^ ions. Concurrently, the introduction of Ag^+^ ion leads to Jahn-Teller distortion, which significantly alters the parity of the wavefunction of self-trapped excitons (STEs), thereby transforming STEs into bright STEs. A hierarchical energy delivery system is identified, which further elevates the photon absorption efficiency and the radiative recombination rate. The ET efficiencies can be calculated for STEs-Mo^4+^ (61.4%), Mo^4+^-Ho^3+^ (17.8%), Mo^4+^-Er^3+^ (15.6%), Mo^4+^-Tm^3+^ (26.4%) and Mo^4+^-Yb^3+^ (39.6%) in In-based system, respectively. These findings underscore the synergistic effects of Mo^4+^/Ag^+^ co-doping in optimizing Ln^3+^-doped NIR luminescent materials. Applicability of In-based and Bi-based systems highlights promising versatile applications. The broadband excitation profile and thermal robustness of these materials make them as prime candidates for NIR pc-LEDs, night vision, imaging, and advanced encryption systems. This work establishes a generalizable framework for overcoming intrinsic limitations of lanthanide-activated luminescence, opening avenues for engineering high-efficiency multifunctional optical materials.

## Materials and methods

### Material

Cesium chloride (CsCl, 99.99%), Silver chloride (AgCl, 99.5%), Bismuth oxide (Bi_2_O_3_, 99.99%), Indium chloride (InCl_3_, 99.99%), Holmium chloride hexahydrate (HoCl_3_·6H_2_O, 99.5%), Erbium chloride hexahydrate (ErCl_3_·6H_2_O, 99.5%), Thulium chloride hexahydrate (TmCl_3_·6H_2_O, 99.99%), Ytterbium chloride hexahydrate (YbCl_3_·6H_2_O, 99.99%), Molybdenum trioxide (MoO_3_, 99.9%) were purchased from Aladdin and Energy Chemical. Molybdenum oxide (MoO_2_, 99%) was purchased from Macklin. Sodium chloride (NaCl, 99.99%), Anhydrous ethanol (AR), and Hydrochloric acid (HCl, 37 wt % in water) were purchased from Sinopharm Chemical Reagent Co., Ltd, China. No additional purification was carried out on the materials and chemicals prior to their use.

### Synthesis of Cs_2_(Na/Ag)InCl_6_:Ln^3+^/Mo^4+^(Ln: Ho, Er, Tm, Yb) crystals

The double perovskite crystals co-doped with Er^3+^ and Mo^4+^ were synthesized via a modified hydrothermal protocol. In brief, a homogeneous precursor solution was formulated by dissolving 2 mmol CsCl, (1-x) mmol NaCl, x mmol AgCl, 1 mmol InCl_3_, stoichiometrically controlled amounts of LnCl_3_·6H_2_O, and MoO_3_ in 10 mL of 6 M HCl within a 25 mL Teflon-lined autoclave. Notably, the dopant incorporation was achieved without altering the stoichiometric ratios of host precursors. The sealed reactor was heated isothermally at 180 °C for 10 h in a program-controlled muffle furnace, followed by a programmed cooling phase (3 °C/h) to ambient temperature to ensure crystalline phase purity. The as-synthesized products were collected by filtration, washed thrice with anhydrous ethanol to remove residual reactants, and dried in a 60 °C oven for 12 h. The acquired crystals were then ground into a fine powder, serving as the basis for subsequent XRD and spectral analyses without further purification. The procedure for synthesizing Cs_2_NaBiCl_6_:Ln^3+^/Mo^4+^ crystals closely mirrored that of Cs_2_(Na/Ag)InCl_6_:Ln^3+^/Mo^4+^ crystals.

### Preparation of the PDMS elastomer

First, the perovskite powder was separately mixed with a PDMS precursor (resin and curing agent weight ratio of 10:1), and the slurries (phosphors and PDMS in a weight ratio of 0.1:1) were filled in the hallow grooves of the pattern. Then, the shallow grooves were heated at 80 °C for 24 h.

### LED fabrication

The NIR phosphor-converted LED devices were fabricated by combining 365 nm or 450 nm chips with Cs_2_NaInCl_6_:Er^3+^/Mo^4+^ sample. Typically, the epoxy resin AB glue was prepared by thoroughly mixing components A and B in a 1:1 mass ratio. Subsequently, the mixed glue was combined with the luminescent material at an equal mass ratio (1:1) and homogenized. The resulting mixture was carefully applied to the LED chip and cured in an oven at 60 °C for 24 h. The cured LEDs were then subjected to various optoelectronic characterization tests and application evaluations. Among them, the commercially available epoxy resin AB can be used, and the LED is bought from Shenzhen looking Long Technology Co., Ltd (3 W, 3.0–3.2 V, ≤700 mA). In addition, the application experiment of the as-fabricated NIR LED penetrating fingers has obtained the informed written consent of all participants.

### NIR penetration and night vision applications

The near-infrared (NIR) penetration experiment involves positioning a palm or an item between NIR camera and NIR LED. When illuminated, the LED emits NIR light which transmits through the palm or item, is detected by the camera, and recorded on a computer. For night vision applications, the object is placed on the desktop with a LED and a NIR camera positioned on opposing sides. Subsequent steps follow the NIR penetration experiment. It is worth noting that 365 nm appears in the experimental photos, 380 and 450 nm are used for luminescence enhancement comparison, and 740 nm only appears in PLQY.

### NIR imaging application

NIR imaging was performed by placing the sample in a 1 mL centrifuge tube, and the centrifuge tube was placed in a dark environment. The tube was then irradiated with a 450 nm laser to excite the sample, inducing NIR emission. The emitted signal was captured using a NIR detector. Power-dependent imaging is to capture the NIR signal emitted by the sample under different power laser irradiation. Penetration depth assessment is to wrap the seal film to the wall of the centrifugal tube, and then measure the thickness of the winding with a vernier caliper (the brand of seal film is parafilm, the model is PM-996, the specification is 28 cm × 38 m).

### Characterizations

Single crystal data collections were recorded using a Bruker Apex II CCD diffractometer with the X-ray Mo Kα radiation (0.71073 Å). Powder X-ray diffraction data were measured using a Bruker D8 Advance X-ray diffraction (XRD) apparatus equipped with the Cu Kα (1.54 Å) radiation at room-temperature. X-ray photoelectron spectroscopy (XPS) measurements were obtained using Thermo Scientific K-Alpha from America. The electron spectrometer uses the Al Kα (1486.6 eV) ray as excitation source in the range 0–1200 eV with a step of 1.0 eV. The Pass Energy is 100 eV for wide spectra, and the Pass Energy is 50 eV for high-resolution XPS analysis. The operating voltage is 12 kV and the filament current is 6 mA. The absorption spectra data was collected using the Hitachi U4150 from Japan. The temperature-dependent and room temperature down-shifting photoluminescence excitation (PLE) and emission (PL) spectra were recorded by a spectrofluorometer with a temperature controller (Edinburgh FLSP-920). Measurements of photoluminescence decay curves and time-resolved PL (TRPL) spectra were performed using the same apparatus with an μF920 lamp as the excitation source. The up-conversion PL spectra and lifetime decay curves were measured with Edinburgh FLSP-920 fluorescence spectrometer equipped with a 980 nm laser. The morphology and elemental composition were obtained by the field-emission scanning electron microscope (FE-SEM, S-4800, Hitachi) equipped with an EDS. The SAED and high-resolution transmission electron microscopes (HRTEM) images were obtained using a FEI Tecnai G2 S-Twin with a field emission gun operating at 200 kV. The element contents were measured by an inductively coupled plasma optical emission spectrometer (ICP-OES) (ICAP 6300, Thermal Scientific). Thermogravimetric analysis (TGA) spectrum was collected with the Perkin-Elmer STA 6000 instrument. The crystals are heated in the range 35–1000 °C at the heating rate of 10 °C per minute, under N_2_ atmosphere. The Raman spectra was collected using the Horiba LabRAM HR Evolution from Japan. The electroluminescence (EL) performance measurements were operated under a voltage of 3 V and currents from 100 to 460 mA on the HAAS 2000 photoelectric measuring system from EVERFINE.

### Photoluminescence lifetime calculation

The photoluminescence (PL) lifetime (*τ*) refers to the time required for luminescent material to return to the ground state by emitting photons after photoexcitation, which is a key parameter characterizing its decay process. The multi-exponential equation was employed to fit the lifetime decay curve:

$${I}_{t}={I}_{0}+{\sum }_{i}{A}_{i}exp\left(\frac{-t}{{\tau }_{i}}\right)$$

where *I*_*t*_ and *I*_*0*_ are the luminescence intensity and the initial intensity at *t* = 0, respectively. *A*_*i*_ is constant, *τ*_*i*_ is the decay time for multi-exponential components and *i* can represent 1 or 2, which means single or double exponential fitting (the STE emission belongs to double exponential fitting and the Mo^4+^ ion emission belongs to single exponential fitting). The average PL lifetime (*τ*_*av*_) is calculated using the equation:

$${\tau }_{{av}}=\frac{{\sum }_{i}{A}_{i}{\tau }_{i}^{2}}{{\sum }_{i}{A}_{i}{\tau }_{i}}$$

### Measurement of photoluminescence quantum yield

The photoluminescence quantum yields (PLQY) of samples were measured by integrating the integrating spheres into the fluorescence spectrophotometer (C9920-2, Hamamatsu Photonics K. K., Japan). The internal quantum efficiency (IQE) is defined as the percentage of the number of emitted photons to that of absorbed photons, which is calculated by the following equation^56,57^:

$${\rm{IQE}}=\frac{\int {L}_{S}}{\int {E}_{R}-\int {E}_{S}}\times 100 \%$$

where *L*_*S*_ is the emission spectrum of the studied sample, *E*_*S*_ is the spectrum of the light used for exciting the sample, and *E*_*R*_ is the spectrum of the excitation light without the sample in the sphere.

The EQE is defined as the percentage of the number of emitted photons to that of excitation photons:

$${\rm{EQE}}=\frac{\int {L}_{S}}{\int {E}_{R}}\times 100 \%$$

The absorption efficiency (Abs) is defined as the percentage of the number of absorbed photons (by the sample) to that of excitation photons:

$${\rm{Abs}}=\frac{\int {E}_{R}-\int {E}_{S}}{\int {E}_{R}}\times 100 \%$$

So the EQE is also calculated using the following equation

$${\mathrm{EQE}}=IQE\times Abs\times 100 \%$$

### The Huang-Rhys factor (*S*) equation

The Huang-Rhys factor (*S*) quantifies the ratio of lattice relaxation energy to phonon energy during electron transition, which is defined as^58^:

$$S=\frac{{\rm{lattice}}\,{\rm{relaxation}}\,{\rm{energy}}}{{\hslash \omega}_{{\rm{phonon}}}}$$

where, the ${\omega}_{{\rm{phonon}}}$ is the phonon frequency. The larger the *S* value, the stronger the interaction between electrons and phonons, resulting in a more significant spectral broadening effect.

The relationship between the full width at half maximum (FWHM), *S*, ${\omega}_{{\rm{phonon}}}$ and temperature (*T*) can be expressed as:

$${\mathrm{FWHM}}=2.36\sqrt{\rm{S}}\cdot \hslash {\omega}\cdot \sqrt{\coth \left(\frac{\hslash {{\upomega }}}{2{\rm{k}}T}\right)}$$

Where k is the Boltzmann constant and *ℏω* is the phonon energy.

Materials are categorized based on *S*:

Weak coupling (*S* ≪ 1): Phonon effects are negligible.

Moderate coupling (*S* = 1–5): Phonons significantly broaden spectral lines.

Strong coupling (*S* ≫ 1): Phonon-assisted transitions dominate.

### Calculation of energy transfer efficiency

The energy transfer efficiency of luminescent materials refers to the efficiency of the excited state energy in the material transferring from donor to acceptor, which is defined as the ratio of the energy received by the acceptor to the initial excitation energy of the donor. It is the core parameter to evaluate the performance of luminescent materials. The energy transfer efficiency (*η*_ET_) is determined by the following equation^59^:

$${\eta }_{ET}=1-\frac{\tau }{{\tau }_{0}}$$

where *τ* and *τ*_*0*_ are the donor lifetimes with and without acceptor, respectively.

### Computational methodology

All DFT calculations were performed using the Vienna Ab Initio Simulation Package^60,61^. The projector augmented-wave method and Perdew-Burke-Ernzerhof generalized gradient approximation (GGA-PBE) were used for the exchange correlation functionals^62,63^. A Monkhorst-Pack k-points mesh with a grid of 3 × 3 × 3 is used for the structure optimizations. The cutoff energy of the plane-wave expansion is set to 450 eV. The convergence threshold for structural optimization was set to be 10^−5 ^eV in energy, and the lattice parameters and internal coordinates are deemed to be fully relaxed when the maximum Hellmann-Feynman force acting on lanthanide ion is less than 0.02 eV Å^−1^.

## Supplementary information

Supplementary information

## Data Availability

The data that support the findings of this study are available within the Article and its Supplementary Information. Related research results are available from the corresponding authors upon reasonable request.
