# Supplementary material for: Sensitizing effect of lanthanide luminescence by Mo4+/Ag+ in double perovskites: great enhancement of near-infrared emission via wide range of excitation (250–850 nm)
Source: Light Sci Appl. 2026 Jan 26;15:87. doi: 10.1038/s41377-025-02159-4 (PMC12835119; doi:10.1038/s41377-025-02159-4)
Supplement: Supplementary file 1 — Supplementary information [file 41377_2025_2159_MOESM1_ESM.pdf]

Supplementary Information for

Sensitizing Effect of Lanthanide Luminescence by Mo^{4+}/Ag^{+} in Double Perovskites: Great Enhancement of Near-Infrared Emission via Wide Range of Excitation (250-850 nm)

Yingsheng Wang,[a,b] Peipei Dang*,[a] Zixun Zeng,[a,b] Dongjie Liu,[a] Guodong Zhang,[c] Long Tian,[a] Kai Li,[a] Ping'an Ma,[a,b] Yi Wei,[d] Hongzhou Lian,[a] Zhiyao Hou,[f] Guogang Li*[d,e] and Jun Lin*[a,b]

a. State Key Laboratory of Rare Earth Resource Utilization, Changchun Institute of Applied Chemistry, Chinese Academy of Sciences, Changchun, Jilin 130022, P. R. China

b. School of Applied Chemistry and Engineering, University of Science and Technology of China, Hefei, Anhui 230026, P. R. China

c. College of Physics Science & Technology, Hebei University, Baoding, Hebei 071002, P. R. China

d. Faculty of Materials Science and Chemistry, China University of Geosciences, Wuhan, Hubei 430074, P. R. China

e. Shenzhen Research Institute, China University of Geosciences, Shenzhen, 518052, P. R. China

f. Guangzhou Municipal and Guangdong Provincial Key Laboratory of Protein Modification and Degradation, School of Basic Medical Sciences, Guangzhou Medical University, Guangzhou, Guangdong 511436, P. R. China

**Correspondence: ppdang@ciac.ac.cn; ggli@cug.edu.cn; jlin@ciac.ac.cn*

Table of Contents

Results and Discussion 4
Table S1. 4
Table S2. 5
Table S3. 6
Table S4. 7
Table S5. 8
Table S6. 9
Table S7. 10
Table S8. 11
Table S9. 12
Table S10. 13
Table S11. 14
Fig. S1 15
Fig. S2 16
Fig. S3 17
Fig. S4 18
Fig. S5 19
Fig. S6 20
Fig. S7 21
Fig. S8 22
Fig. S9 23
Fig. S10 24
Fig. S11 25
Fig. S12 26
Fig. S13 27
Fig. S14 28
Fig. S15 29
Fig. S16 30
Fig. S17 31
Fig. S18 32
Fig. S19 33
Fig. S20 34
Fig. S21 35
Fig. S22 36
Fig. S23 37
Fig. S24 38
Fig. S25 39
Fig. S26 40
Fig. S27 41
Fig. S28 42
Fig. S29 43
Fig. S30 44

Fig. S31 45
Fig. S32 46
Fig. S33 47
Fig. S34 48
Fig. S35 49
Fig. S36 50
Fig. S37 51
References 52

Results and Discussion

Table S1. Structural parameters of single crystals through SC-XRD analysis

Structural parameter	$Cs_2NaInCl_6$	$Cs_2NaInCl_6$:Mo^{4+}	$Cs_2NaInCl_6$:Mo^{4+}/Er^{3+}
Temperature	293 K	293 K	293 K
$a = b = c$	10.5360(9) Å	10.5168(10) Å	10.5190(5) Å
$\alpha = \beta = \gamma$	90°	90°	90°
volume	1169.6(3) Å^3	1163.2(3) Å^3	1163.92(17) Å^3
Space group	Fm-3m	Fm-3m	Fm-3m
Z	4	4	4
Dx	3.500 g cm^{-3}	3.519 g cm^{-3}	3.517 g cm^{-3}
h, k, lmax	15, 15, 15	16, 15, 15	17, 17, 17
R(reflections)	0.0165(121)	0.0136(150)	0.0120(172)
wR_2(reflections)	0.0328(124)	0.0258(155)	0.0250(177)

Table S2. Crystallographic parameters obtained from XRD Rietveld refinements

Sample	Space group	Crystallographic parameters		Reliability factors		
		$a = b = c$ (Å)	V (Å^3)	R_p	R_{wp}	χ^2
Host	Cubic Fm-3m	10.5336	10.5336	10.5336	1168.759	5.77%
NIE		10.5367	10.5367	10.5367	1169.792	9.31%
NIME		10.5317	10.5317	10.5317	1168.158	8.81%
NAIME		10.5255	10.5255	10.5255	1166.083	8.89%

Table S3. Bond length values obtained from XRD Rietveld refinements

Sample	Bond	Length (Å)
Host	Na-Cl	2.731(1)
	In-Cl	2.536(1)
NIE	Na-Cl	2.702(20)
	In-Cl	2.566(20)
NIME	Na-Cl	2.726(18)
	In-Cl	2.540(18)
NAIME	Na-Cl	2.736(20)
	In-Cl	2.527(20)

Table S4. The actual doping ion concentration of the studied samples by using ICP-OES measurement

Sample	Precursor			Product		
	Ag/%	Mo/%	Er/%	Ag/%	Mo/%	Er/%
NIE	0	0	30	0	0.00	1.26
NIM	0	20	0	0	2.64	0.00
NIME	0	20	30	0	2.89	1.13
NAIME	20	20	30	1	2.75	1.04

Table S5. Comparison of luminescent properties for some typical Ln^{3+} ions doped NIR lead-free metal halide materials

	Sample	PLE/nm	PL/nm	PLQY (λ_{ex})	Ref
1	$Cs_2AgInCl_6:Na^+/Bi^{3+}/Nd^{3+}$	250-450	800-1400	30.3 % (370 nm)	1
2	$Cs_2NaInCl_6:Sb^{3+}/Tm^{3+}$	300-400	1100-1300	58.0 % (320 nm)	2
3	$Cs_2AgInCl_6:Cr^{3+}/Yb^{3+}$	300-450, 500-900	800-1400	45.0 % (365 nm)	3
4	$Cs_2Ag_{1-x}Na_xBiCl_6:Yb^{3+}$	200-450	900-1050	19.0 % (360 nm)	4
5	$Cs_2NaScCl_6:Er^{3+}$	250-550	1400-1650	28.3 % (380 nm)	5
6	$Cs_2NaInCl_6:Yb^{3+}$	200-400	900-1100	39.4 % (273 nm)	6
7	$Cs_2Ag_{0.8}Na_{0.2}InCl_6:Mn^{2+}/Yb^{3+}/Er^{3+}$	250-500	900-1300	28.0 % (365 nm)	7
8	$Cs_2AgInCl_6:Cr^{3+}/Er^{3+}$	250-450, 500-700, 700-900	800-1400, 1400-1600	57.5 % (356 nm)	8
9	$Cs_2NaGdCl_6:Yb^{3+}/Er^{3+}$	250-400	800-1100	29.0 % (378 nm)	9
10	$Cs_4MnBi_2Cl_{12}:RE^{3+}$	250-600	800-1600	59.0 % (370 nm)	10
11	$Cs_2NaYCl_6:Sb^{3+}/Tm^{3+}$	260-380	1100-1300	20.2 % (320 nm)	11
12	$Cs_2ZrCl_6:Te^{4+}/Er^{3+}$	220-500	1450-1600	6.1 % (392 nm)	12
13	$Cs_2NaInCl_6:Sb^{3+}/Yb^{3+}$	250-400	900-1100	49.0 % (318 nm)	13
14	$Cs_2NaBi_{1-x}Er_xCl_6:Mn^{2+}$	250-550	1450-1600	14.2 % (360 nm)	14
15	$Cs_2KYbCl_6:Sb^{3+}$	250-450	900-1100	43.8 % (345 nm)	15
16	$Cs_2NaYbCl_6:Sb^{3+}$	300-450	900-1100	56.8 % (350 nm)	16
17	$Cs_2NaYCl_6:Er^{3+}$	250-600	1450-1600	87 % (520 nm)	17
18	$Cs_2NaLuCl_6:Cr^{3+}$	250-450, 500-800,	800-1400	92.9 % (300 nm)	18
19	$Cs_2Ag_{0.6}Na_{0.4}In_{0.9}Bi_{0.1}Cl_6:Tm^{3+}$	300-450	750-900	22.7 % (405 nm)	19
20	$Cs_2NaLuCl_6:Sb^{3+}/Er^{3+}/Yb^{3+}$	250-450	1450-1600	37.1 % (365 nm)	20
21	$Cs_2(Na/Ag)InCl_6:Mo^{4+}/Er^{3+}$	250-850	750-1400, 1400-1600	≈100 % (460 nm)	This work

Table S6. The bond length values obtained by structural optimization calculation

Sample	Bond	Length (Å)
NIE	Na1-Cl1	2.657(0)
	Na1-Cl2	2.731(0)
	Na2-Cl	2.721(0)
	In-Cl1	2.537(0)
	In-Cl2	2.547(0)
	Er-Cl	2.611(0)
NIME	Na-Cl1	2.618(0)
	Na-Cl2	2.735(0)
	Na-Cl3	2.848(0)
	In-Cl1	2.533(0)
	In-Cl2	2.566(0)
	Mo-Cl1	2.420(0)
	Mo-Cl2	2.428(0)
	Mo-Cl3	2.441(0)
	Er-Cl1	2.650(0)
	Er-Cl2	2.658(0)
NAIME	Na1-Cl1	2.623(0)
	Na1-Cl2	2.734(0)
	Na1-Cl3	2.843(0)
	Na2-Cl1	2.699(0)
	Na2-Cl2	2.826(0)
	Ag-Cl1	2.562(0)
	Ag-Cl2	2.844(0)
	In-Cl1	2.534(0)
	In-Cl2	2.569(0)
	Mo-Cl1	2.424(0)
	Mo-Cl2	2.425(0)
	Mo-Cl3	2.442(0)
	Er-Cl1	2.645(0)
	Er-Cl2	2.706(0)

Table S7. The luminescence lifetimes and calculated ET efficiencies from STE to Mo^{4+} at various concentrations of Mo^{4+} in $Cs_2(Na/Ag)InCl_6$:Mo^{4+} system

Concentration (Mo^{4+})	Lifetime (STE, μs)	η_{ET} (STE-Mo^{4+}, %)
0	2.53	-
0.1	1.96	22.6
0.2	1.48	41.5
0.3	1.23	51.5
0.4	0.98	61.4

Table S8. The luminescence lifetimes and calculated ET efficiencies from Mo^{4+} to Ho^{3+} at various concentrations of Ho^{3+} in $Cs_2NaInCl_6$:Mo^{4+}/Ho^{3+} system

Concentration (Ho^{3+})	Lifetime (Mo^{4+}, μs)	η_{ET} (Mo^{4+}-Ho^{3+}, %)
0	59.3	-
0.1	55.3	6.8
0.2	51.1	13.8
0.3	50.1	15.4
0.4	48.7	17.8

Table S9. The luminescence lifetimes and calculated ET efficiencies from Mo^{4+} to Er^{3+} at various concentrations of Er^{3+} in $Cs_2NaInCl_6$:Mo^{4+}/Er^{3+} system

Concentration (Er^{3+})	Lifetime (Mo^{4+}, μs)	η_{ET} (Mo^{4+}-Er^{3+}, %)
0	54.2	-
0.1	49.6	8.4
0.2	48.4	10.7
0.3	45.9	15.3
0.4	45.7	15.6

Table S10. The luminescence lifetimes and calculated ET efficiencies from Mo^{4+} to Tm^{3+} at various concentrations of Tm^{3+} in $Cs_2NaInCl_6$:Mo^{4+}/Tm^{3+} system

Concentration (Tm^{3+})	Lifetime (Mo^{4+}, μs)	η_{ET} (Mo^{4+}-Tm^{3+}, %)
0	59.3	-
0.1	51.1	13.9
0.2	48.3	18.6
0.3	47.5	20.0
0.4	43.7	26.4

Table S11. The luminescence lifetimes and calculated ET efficiencies from Mo^{4+} to Yb^{3+} at various concentrations of Yb^{3+} in $Cs_2NaInCl_6$:Mo^{4+}/Yb^{3+} system

Concentration (Yb^{3+})	Lifetime (Mo^{4+}, μs)	η_{ET} (Mo^{4+}-Yb^{3+}, %)
0	59.3	-
0.1	45.1	23.9
0.2	43.5	26.6
0.3	39.4	33.6
0.4	35.8	39.6

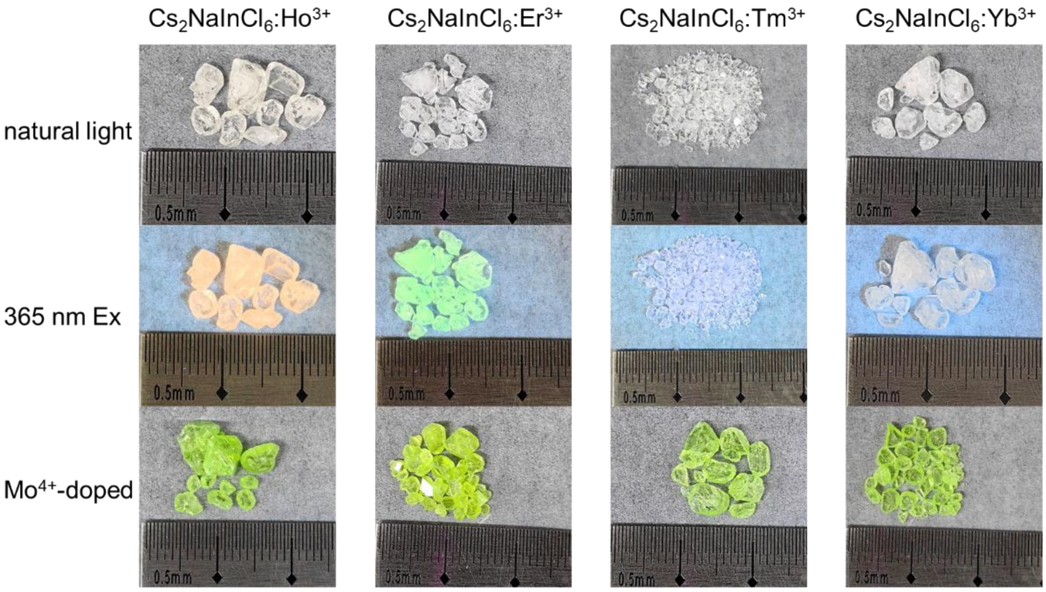

Fig. S1 The photographs of Ln^{3+}- and Mo^{4+}/Ln^{3+}-doped $Cs_2NaInCl_6$ samples.

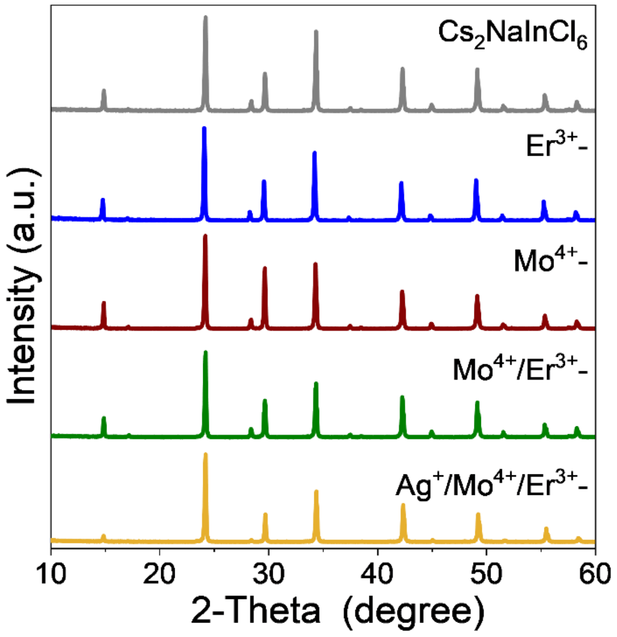

Fig. S2 XRD patterns of host and Mo^{4+}/Er^{3+}-doped $Cs_2NaInCl_6$ samples.

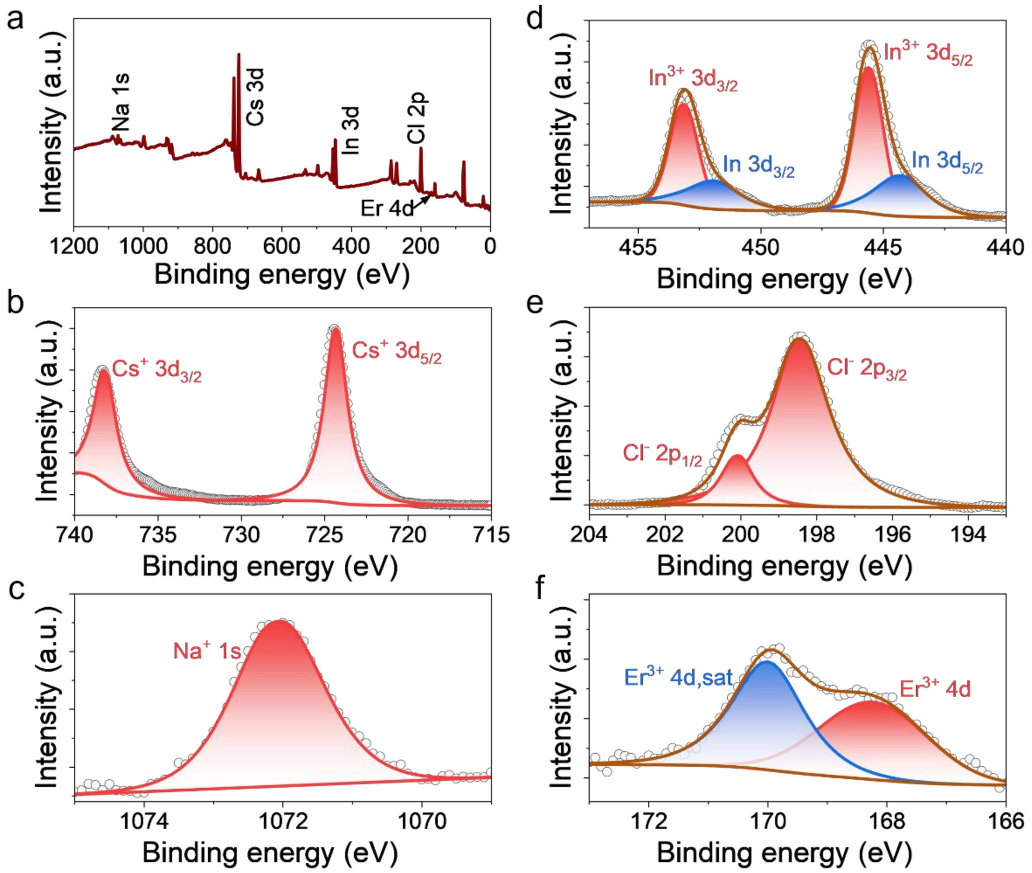

Fig. S3 a) The XPS survey analysis of $Cs_2NaInCl_6:Er^{3+}$. b-f) The high-resolution XPS analysis of Cs 3*d*, Na 1*s*, In 3*d*, Cl 2*p* and Er 4*d*, respectively.

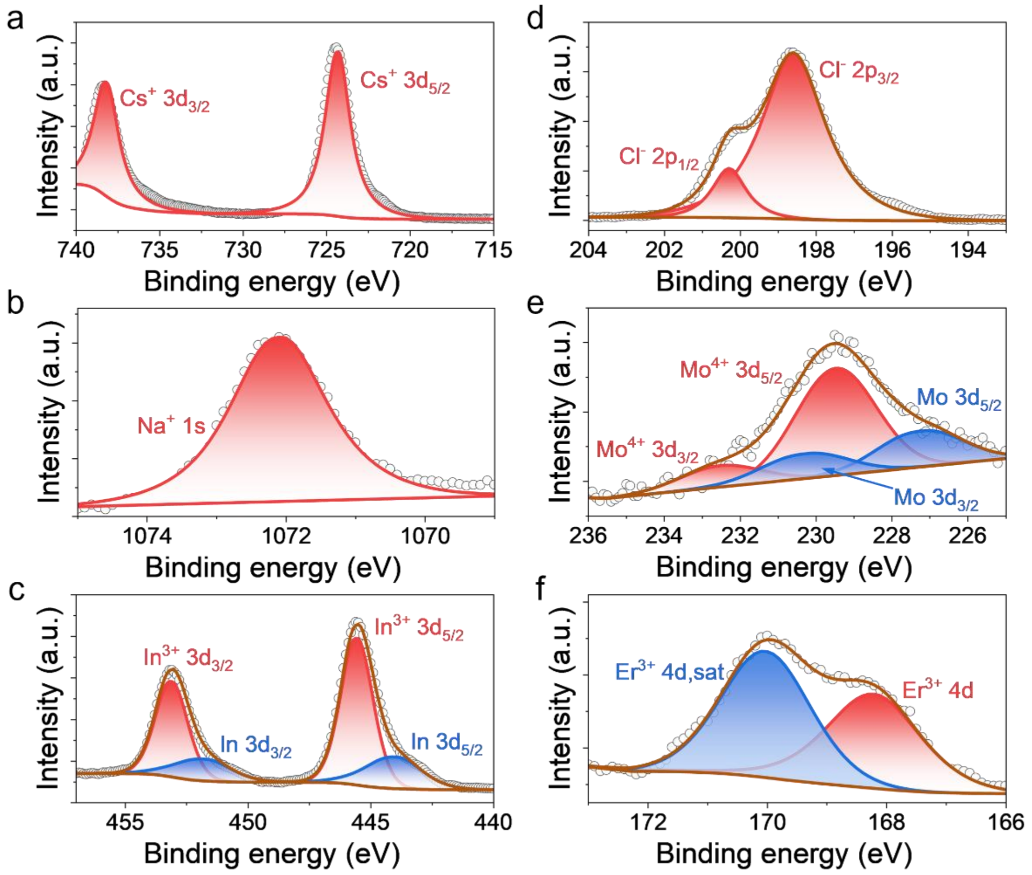

Fig. S4 The high-resolution XPS analysis of Cs 3*d*, Na 1*s*, In 3*d*, Cl 2*p*, Mo 3*d* and Er 4*d* in $Cs_2NaInCl_6$:Mo^{4+}/Er^{3+}, respectively.

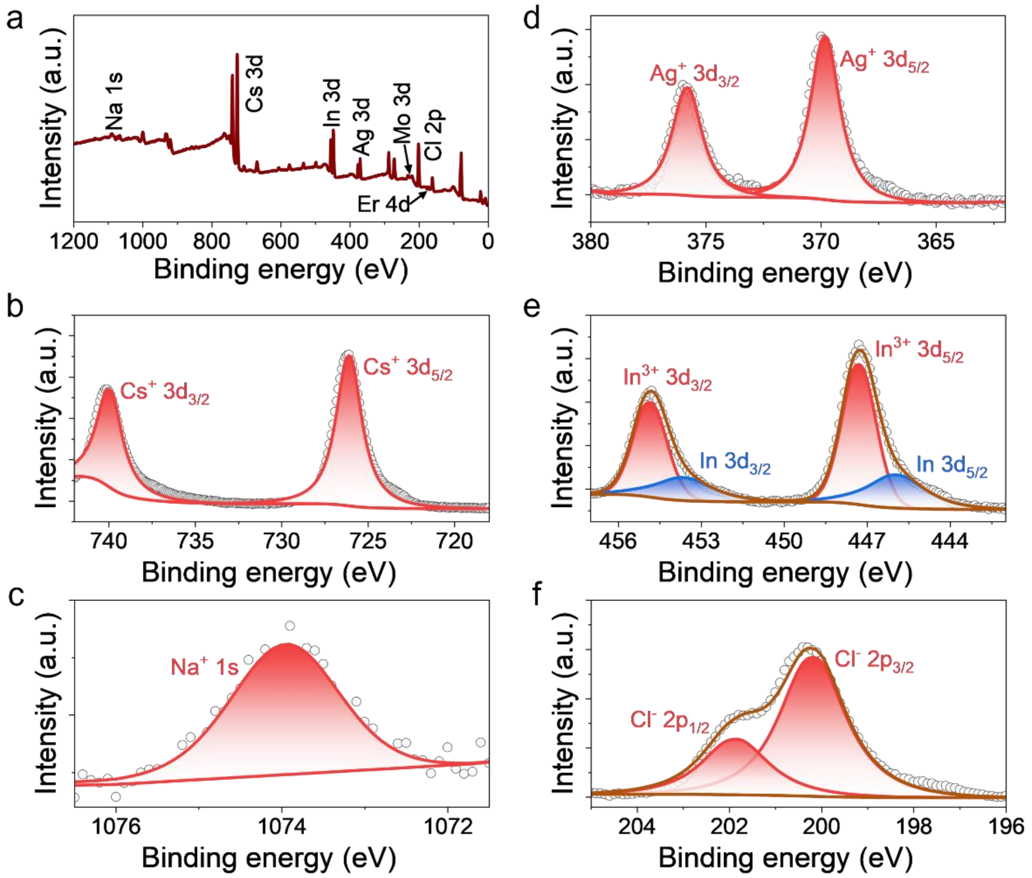

Fig. S5 a) The XPS survey analysis of $Cs_2(Na/Ag)InCl_6$:Mo^{4+}/Er^{3+}. b-f) The high-resolution XPS analysis of Cs 3*d*, Na 1*s*, Ag 3*d*, In 3*d* and Cl 2*p*, respectively.

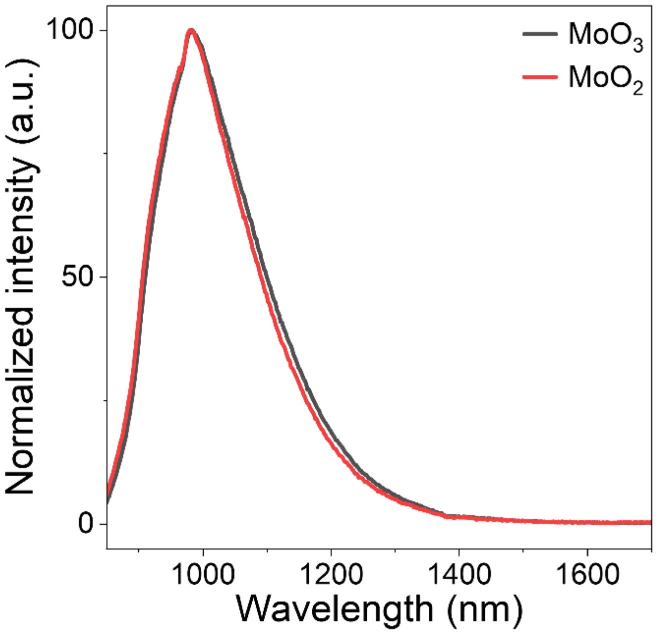

Fig. S6 The emission spectra of $Cs_2NaInCl_6$:Mo^{4+} synthesized with MoO_2 and MoO_3 as raw materials, respectively.

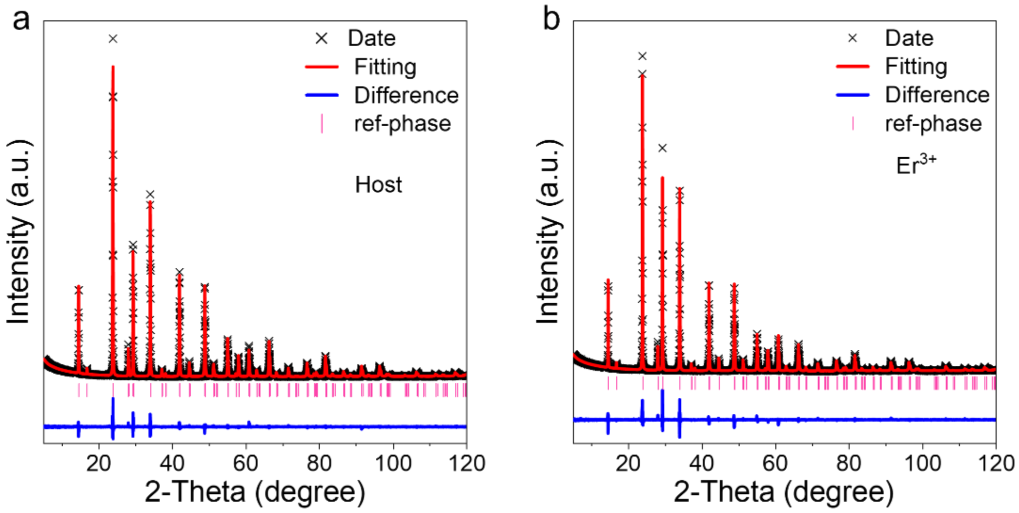

Fig. S7 XRD Rietveld refinement of the as-prepared a) $Cs_2NaInCl_6$ and b) $Cs_2NaInCl_6:Er^{3+}$ samples.

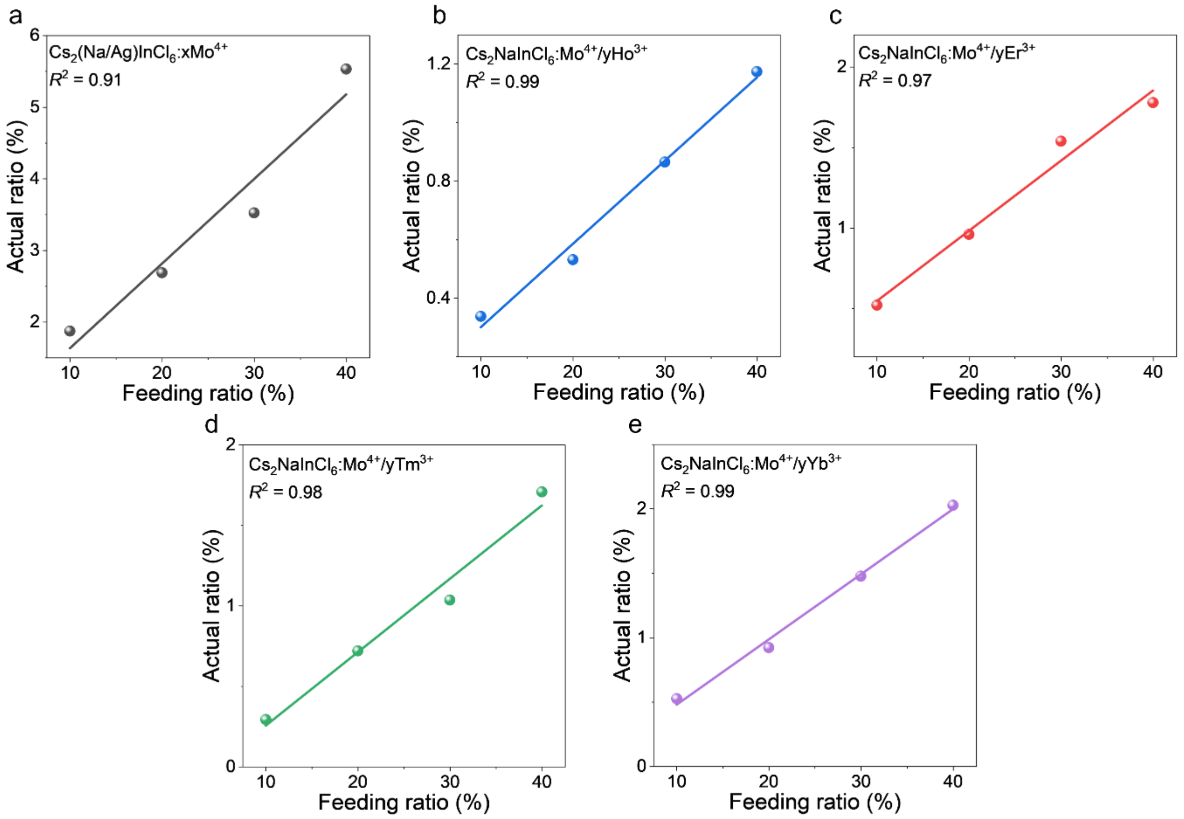

Fig. S8 The actual doping ion concentration of Mo^{4+}, Ho^{3+}, Er^{3+}, Tm^{3+} and Yb^{3+} by using ICP-OES measurement, respectively.

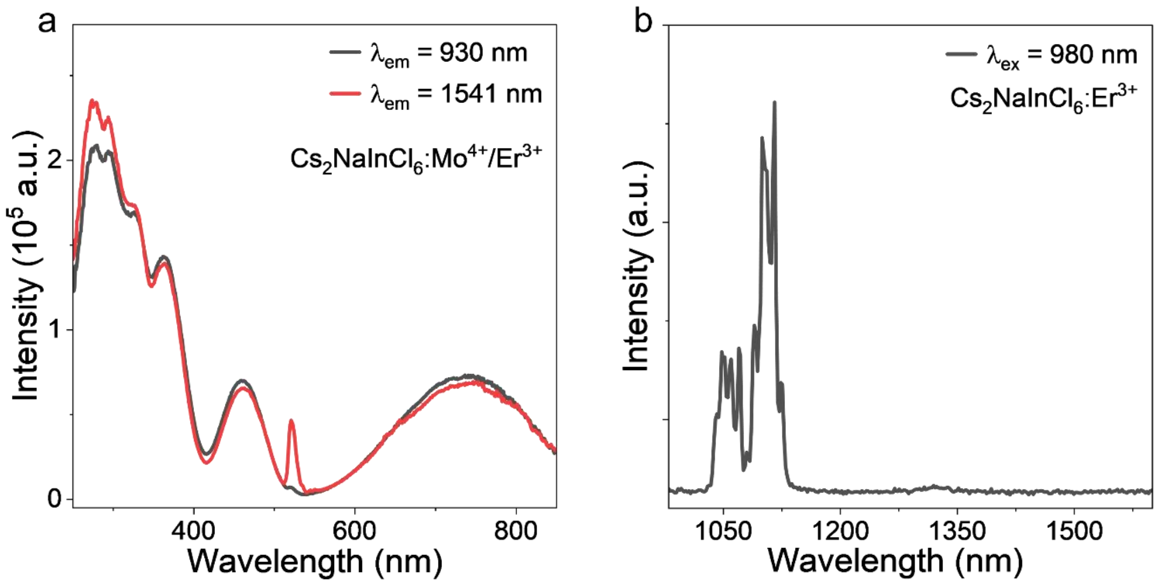

Fig. S9 a) The PLE spectra of $Cs_2NaInCl_6$:Mo^{4+}/Er^{3+} by detecting 930 and 1541 nm respectively. b) the PL spectrum of $Cs_2NaInCl_6$:Er^{3+} (λ_{ex} = 980 nm).

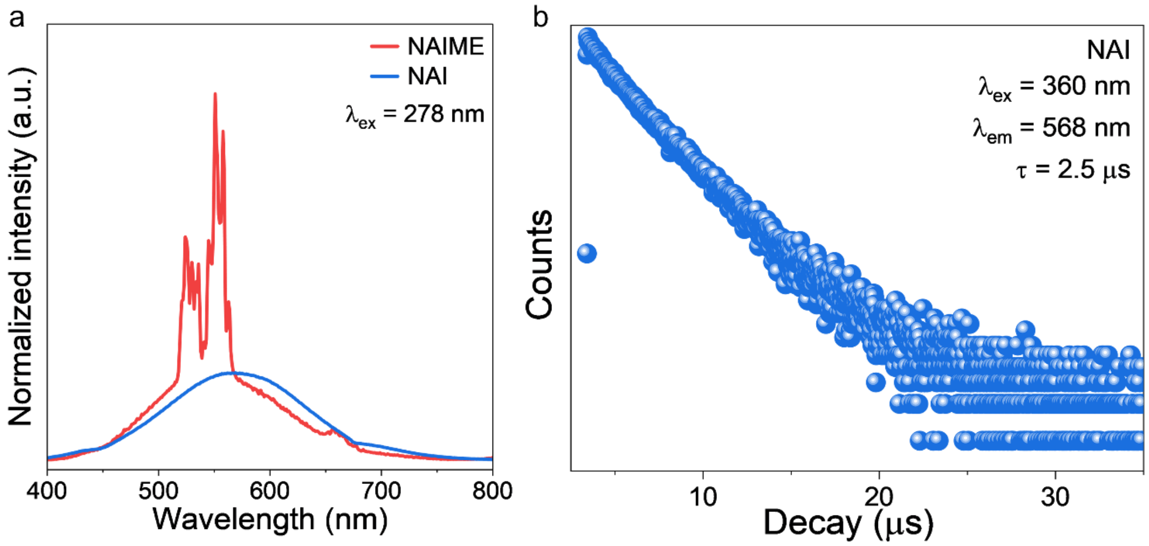

Fig. S10 a) The visible PL spectra of $Cs_2(Na/Ag)InCl_6{:}Mo^{4+}/Er^{3+}$ and $Cs_2(Na/Ag)InCl_6$ (λ_{ex} = 278 nm), b) the luminescence decay curve of $Cs_2(Na/Ag)InCl_6$ measured at 568 nm under 360 nm excitation.

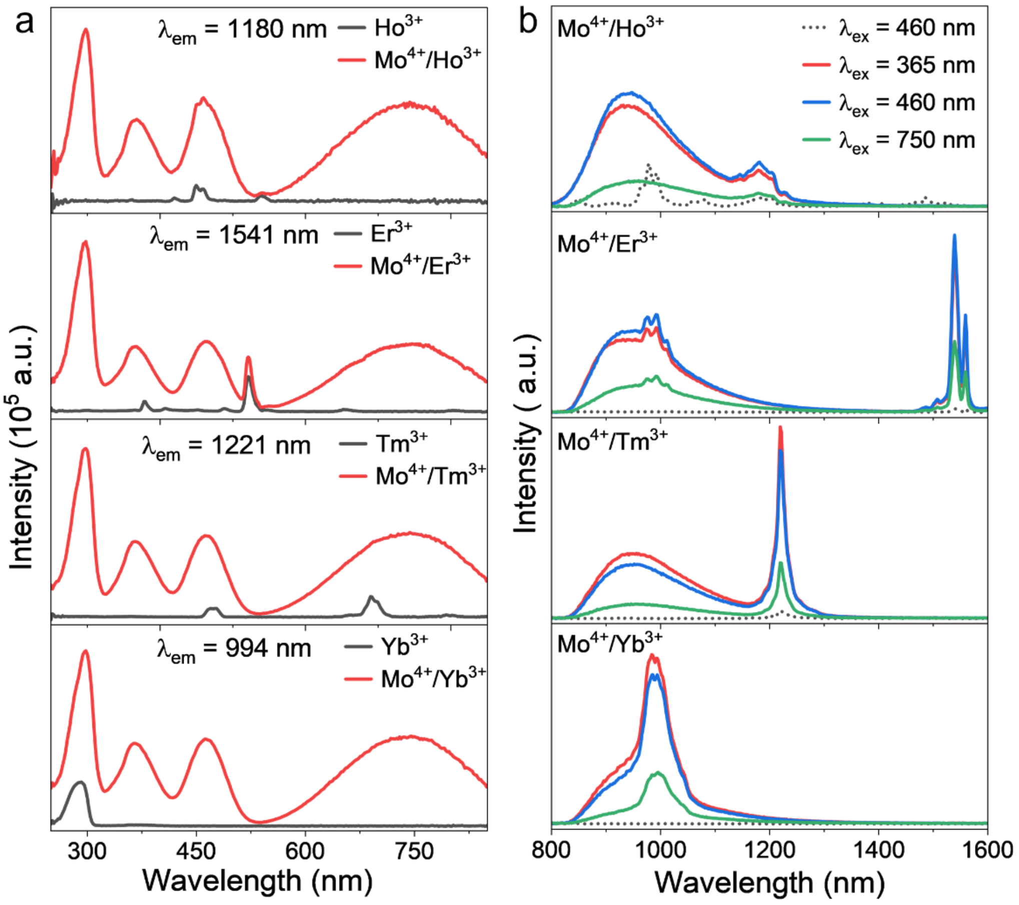

Fig. S11 a) PLE and b) PL spectra of $Cs_2NaBiCl_6$:Mo^{4+}/Ln^{3+} (Ln: Ho, Er, Tm, Yb) under different excitation (the excitation conditions of $Cs_2NaInCl_6$:Mo^{4+}/Ln^{3+} are consistent, and the grey dotted lines are the PL spectra of Ln^{3+} singly doped materials) and emission wavelengths. The PLE spectra of Mo^{4+} ions are different due to the different hosts.

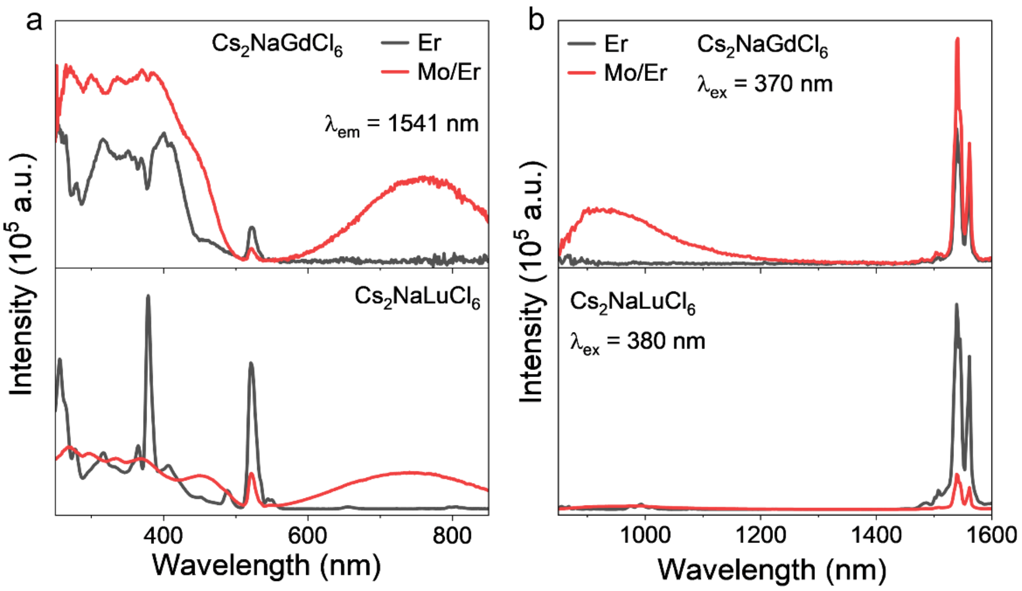

Fig. S12 The a) PLE and b) PL spectra of Cs_2NaMCl_6:Mo^{4+}/Er^{3+} (M: Gd, Lu).

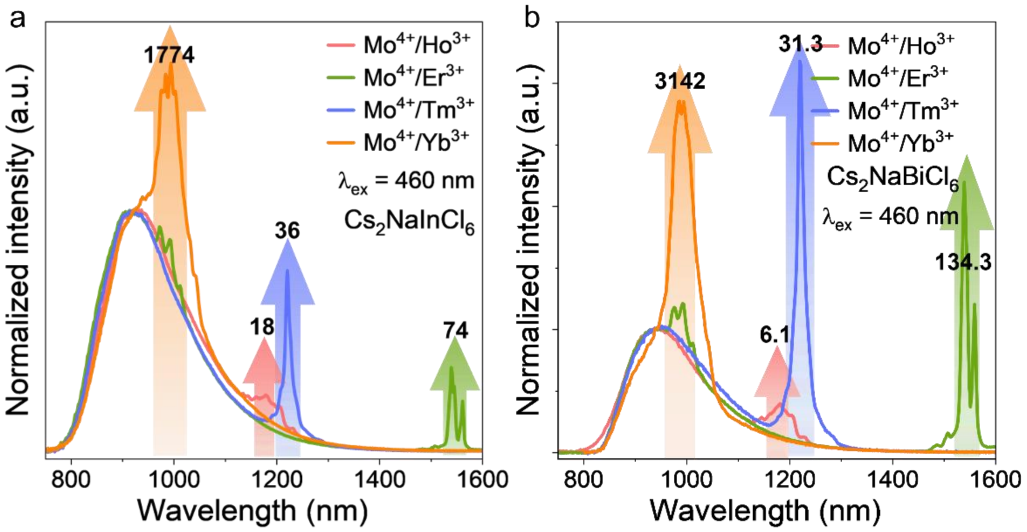

Fig. S13 Normalized PL spectra of Mo^{4+}/Ln^{3+} co-doped a) $Cs_2NaInCl_6$ and b) $Cs_2NaBiCl_6$ (Ln: Ho, Er, Tm, Yb) samples, the enhancement factors are derived from the calculation of Fig. S11 and Fig. S12, respectively.

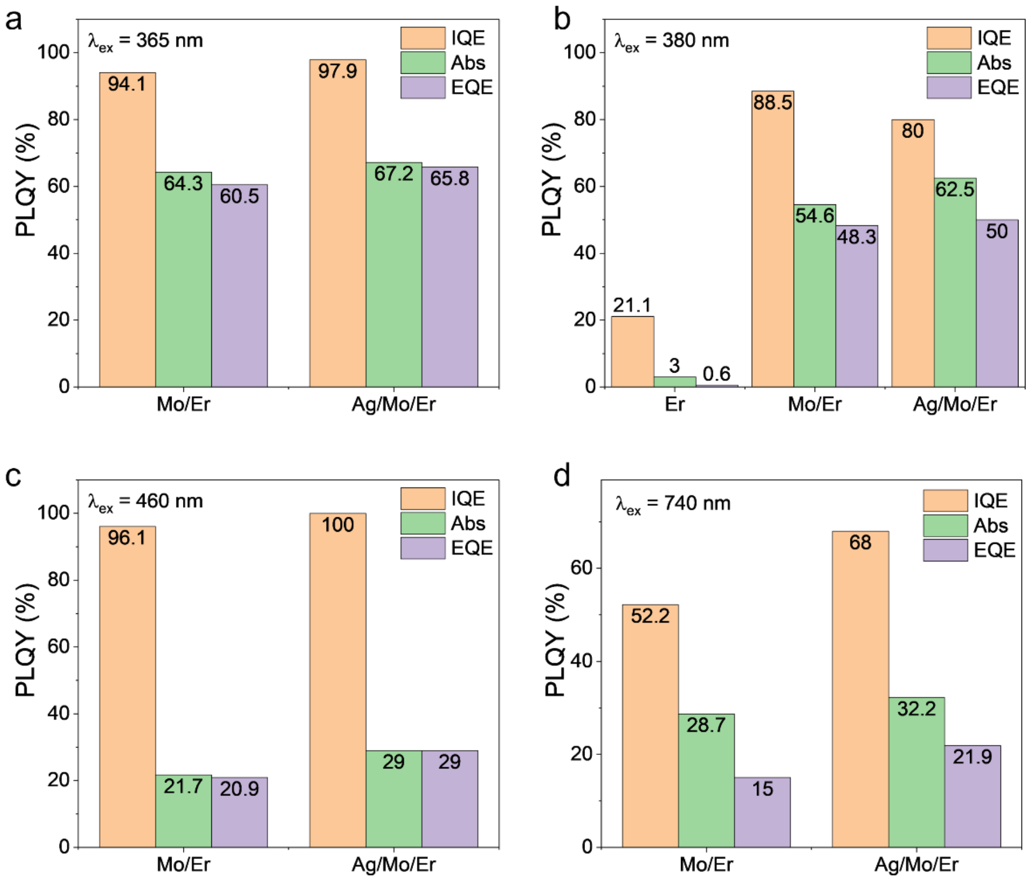

Fig. S14 Abs, IQE and EQE values of $Cs_2NaInCl_6:Er^{3+}$, $Cs_2NaInCl_6:Mo^{4+}/Er^{3+}$and $Cs_2(Na/Ag)InCl_6:Mo^{4+}/Er^{3+}$ under the excitation of a) 365 nm, b) 380 nm, c) 460 nm and d) 740 nm light.

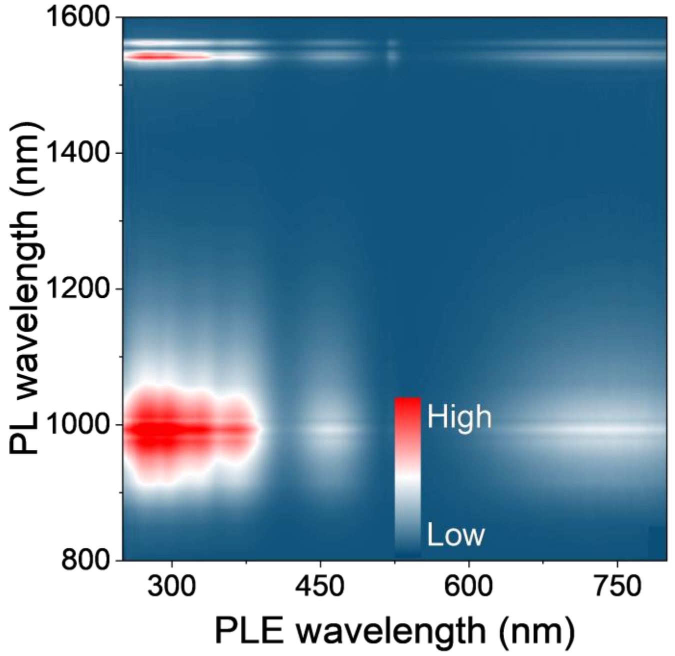

Fig. S15 Emission-dependent PLE spectra of $Cs_2NaInCl_6$:Mo^{4+}/Er^{3+}.

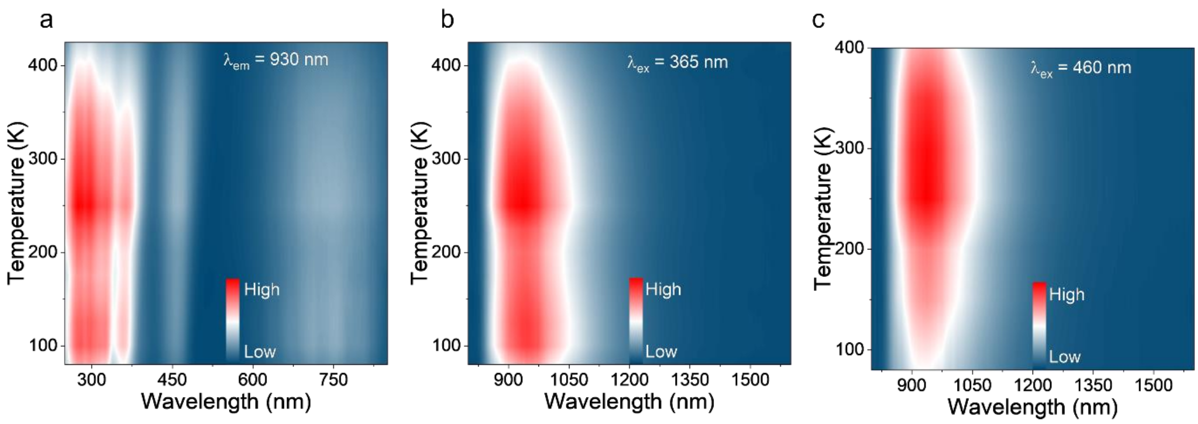

Fig. S16 a) Temperature-dependent PLE spectra of $Cs_2NaInCl_6$:Mo^{4+} (λ_{em} = 930 nm). b-c) Temperature-dependent PL spectra of $Cs_2NaInCl_6$:Mo^{4+} (λ_{ex} = 365, 460 nm).

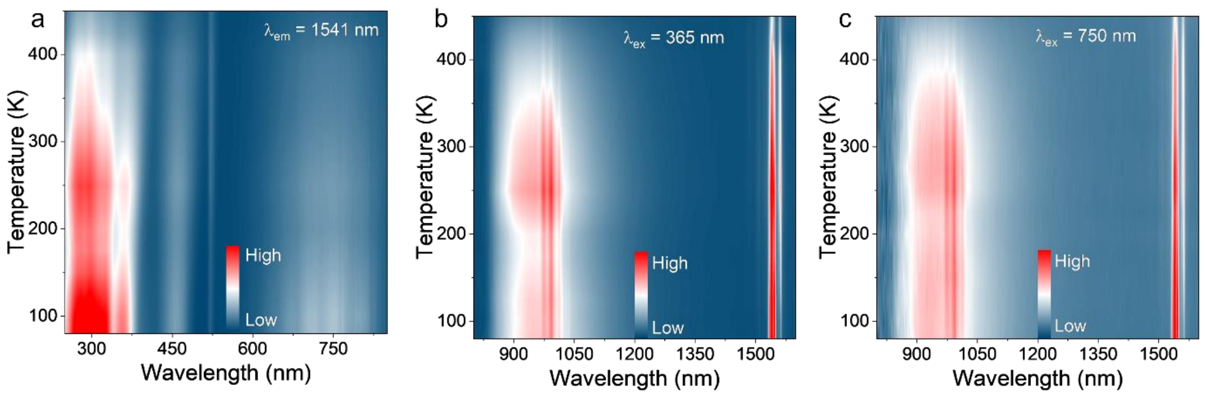

Fig. S17 a) Temperature-dependent PLE spectra of $Cs_2NaInCl_6:Mo^{4+}/Er^{3+}$ (λ_{em} = 1541 nm). b-c) Temperature-dependent PL spectra of $Cs_2NaInCl_6:Mo^{4+}/Er^{3+}$ (λ_{ex} = 365, 750 nm).

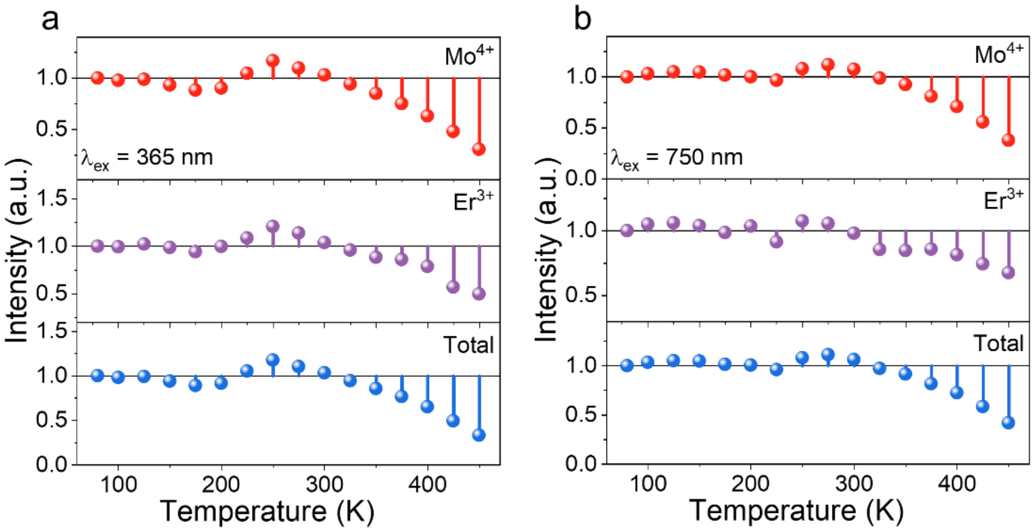

Fig. S18 The change of temperature-dependent PL intensity of $Cs_2NaInCl_6:Mo^{4+}/Er^{3+}$.

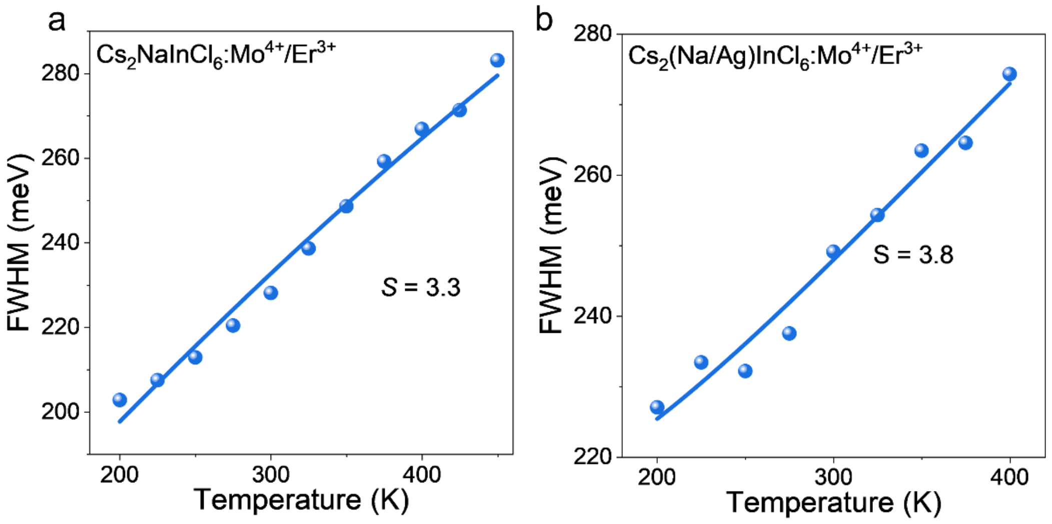

Fig. S19 The calculation of Huang-Rhys factor for a) $Cs_2NaInCl_6:Mo^{4+}/Er^{3+}$ and b) $Cs_2(Na/Ag)InCl_6:Mo^{4+}/Er^{3+}$.

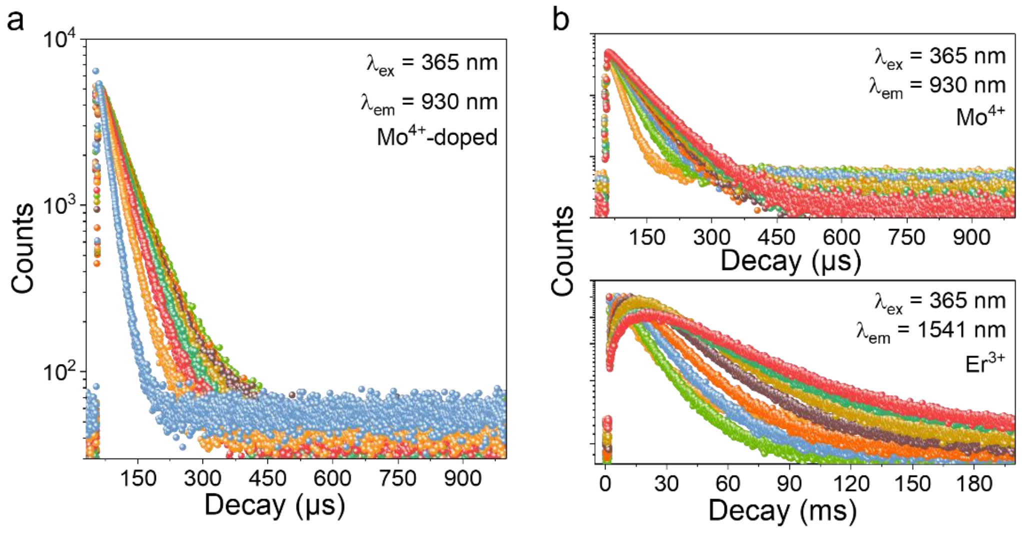

Fig. S20 Temperature-dependent lifetime profile changes in $Cs_2NaInCl_6:Mo^{4+}$ and $Cs_2NaInCl_6:Mo^{4+}/Er^{3+}$, respectively.

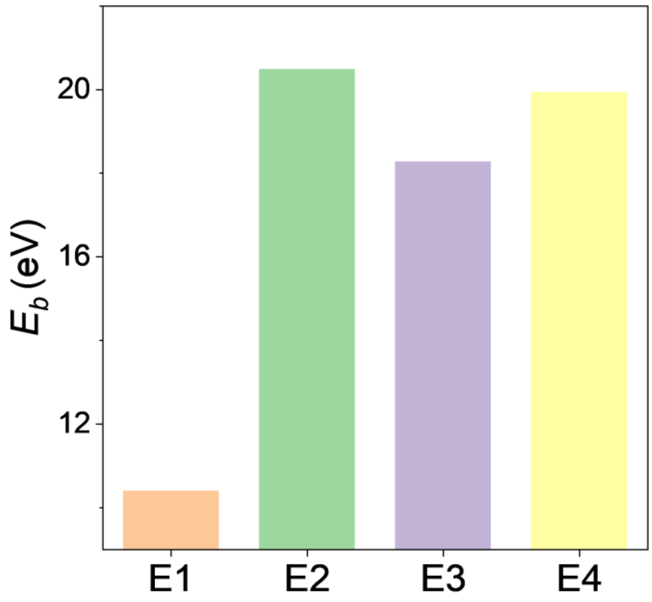

Fig. S21 The binding energies of four possible configurations (E1: Na_{Mo}, E2: In_{Mo}, E3:V_{In}+Na_{Mo}, E4: V_{Na}+In_{Mo}).

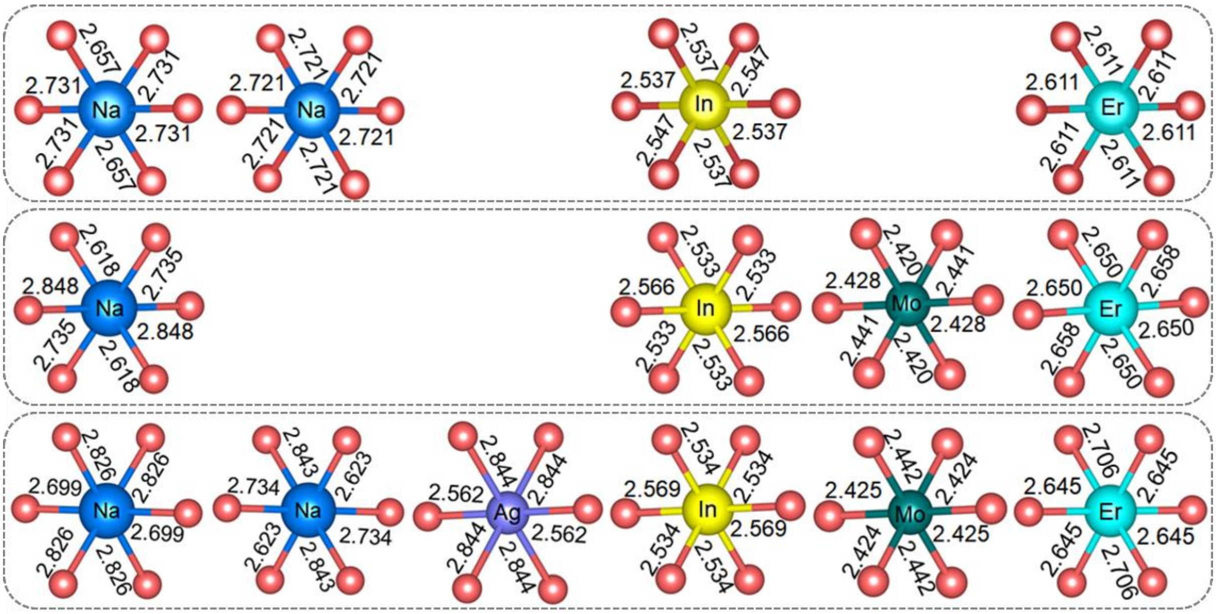

Fig. S22 The octahedrons drawn by structural optimization calculation (The first line is $Cs_2NaInCl_6$:Er^{3+}, the second line is $Cs_2NaInCl_6$:Mo^{4+}/Er^{3+}, and the third line is $Cs_2(Na/Ag)InCl_6$:Mo^{4+}/Er^{3+}).

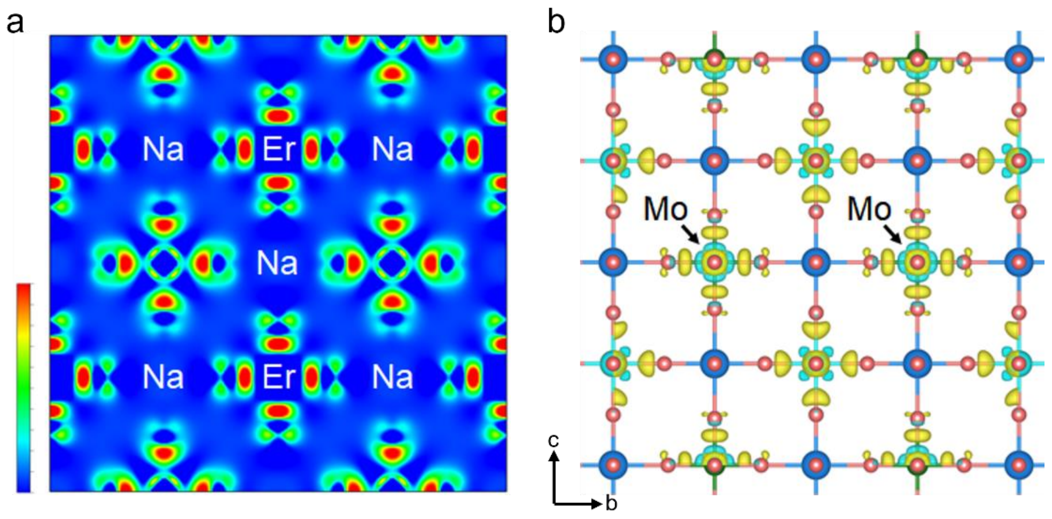

Fig. S23 a) The charge density distribution and b) the differential charge distribution of $Cs_2NaInCl_6$:Mo^{4+}/Er^{3+}.

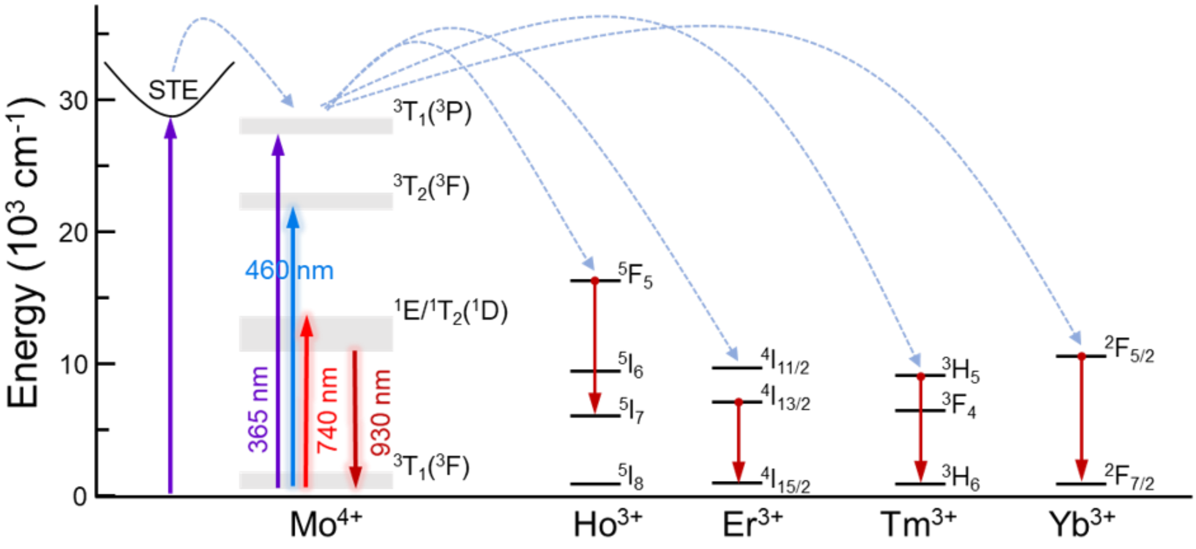

Fig. S24 Proposed mechanism of efficient sensitization.

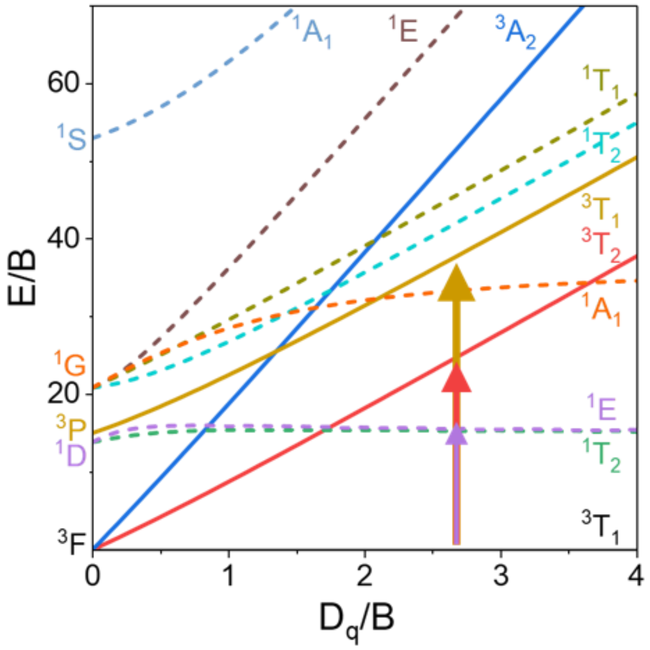

Fig. S25 Tanabe-Sugano diagram for d^2 octahedral system.

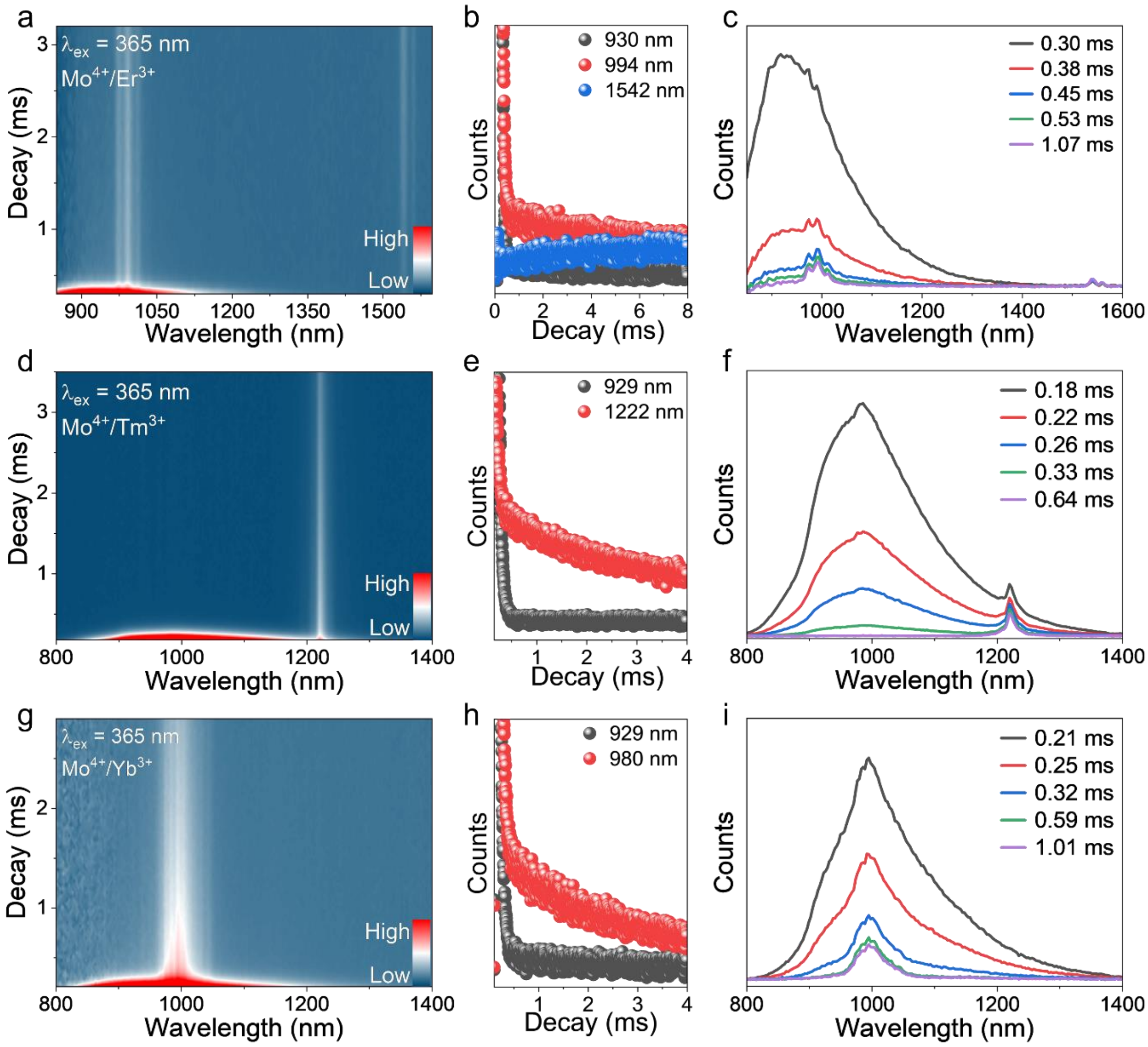

Fig. S26 a), d) and g) Time-resolved PL spectra of $Cs_2NaInCl_6:Mo^{4+}/Er^{3+}$, $Cs_2NaInCl_6:Mo^{4+}/Tm^{3+}$ and $Cs_2NaInCl_6:Mo^{4+}/Yb^{3+}$ under 365 nm excitation, respectively; b), e) and h) TRPL kinetics analysis of $Cs_2NaInCl_6:Mo^{4+}/Er^{3+}$, $Cs_2NaInCl_6:Mo^{4+}/Tm^{3+}$ and $Cs_2NaInCl_6:Mo^{4+}/Yb^{3+}$ at different characteristic emission peaks, respectively; c), f) and i) TRPL spectra of $Cs_2NaInCl_6:Mo^{4+}/Er^{3+}$, $Cs_2NaInCl_6:Mo^{4+}/Tm^{3+}$ and $Cs_2NaInCl_6:Mo^{4+}/Yb^{3+}$ at different time delays, respectively.

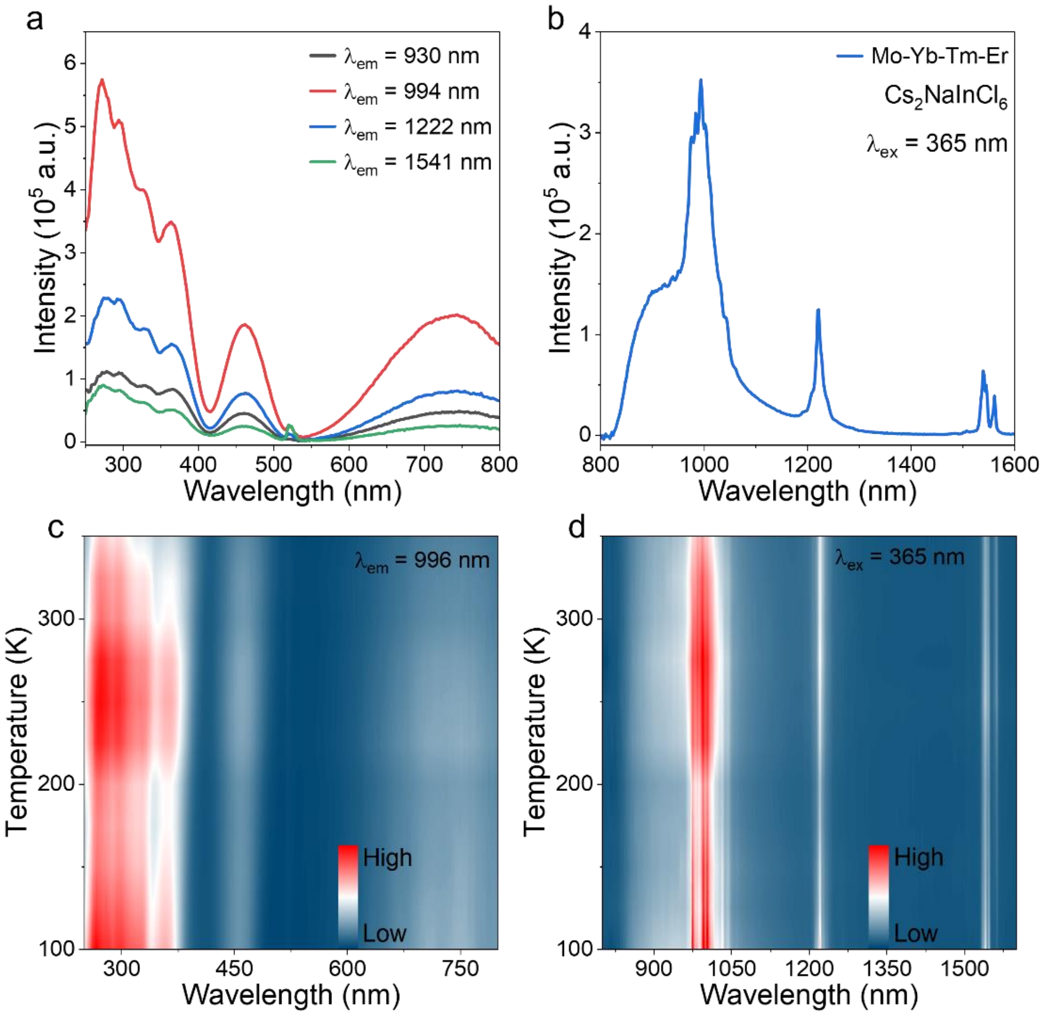

Fig. S27 $Cs_2NaInCl_6:Mo^{4+}/Ln^{3+}$ (Ln: Yb, Tm, Er): a) PLE and b) PL spectra at RT, and temperature-dependent c) PLE and d) PL spectra at 100-350 K.

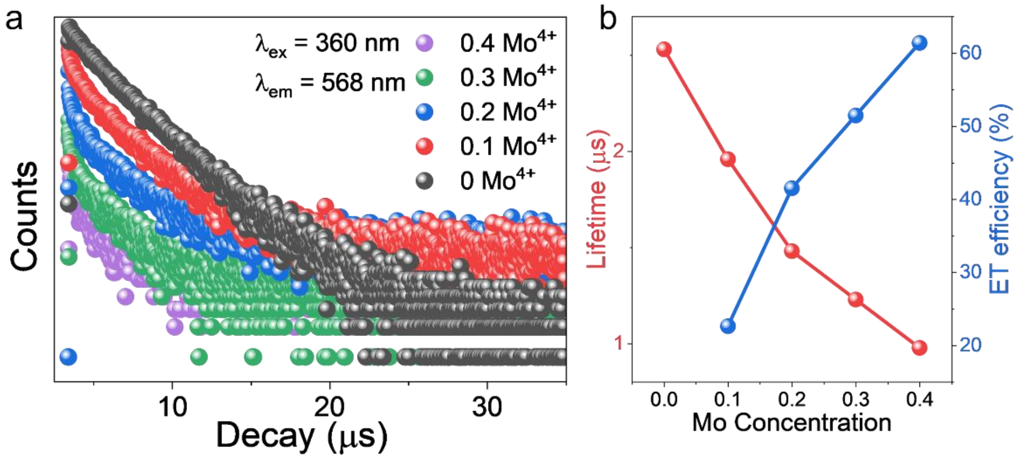

Fig. S28 a) Luminescence decay curves of $Cs_2(Na/Ag)InCl_6$:Mo^{4+} measured at 568 nm with different Mo^{4+} doping concentration under 360 nm excitation. b) The lifetime values and ET efficiencies of $Cs_2(Na/Ag)InCl_6$:Mo^{4+} from STE to Mo^{4+} in different Mo^{4+} doping concentration.

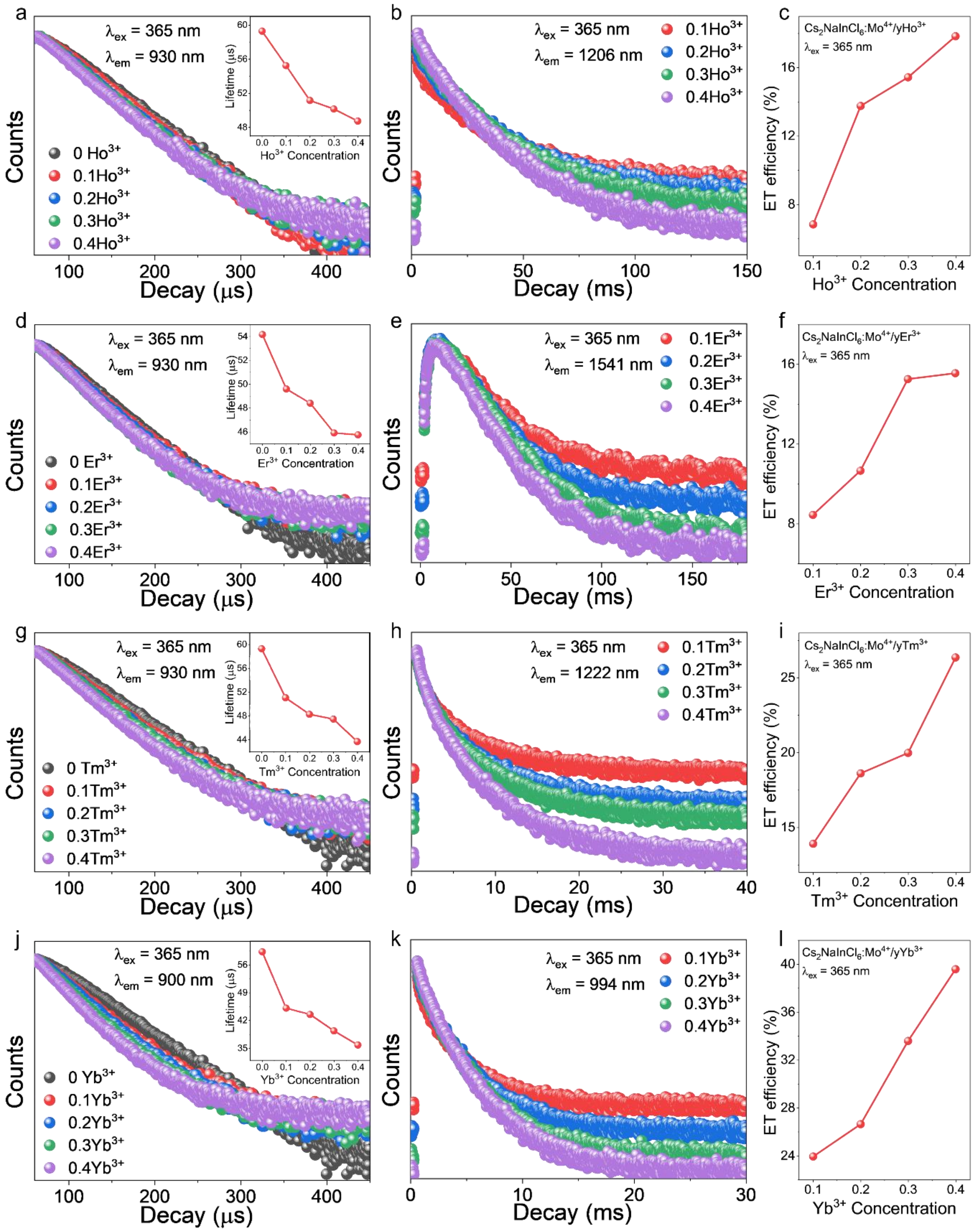

Fig. S29 a), d), g) and j) Luminescence decay curves of $Cs_2NaInCl_6$:Mo^{4+}/Ho^{3+}, $Cs_2NaInCl_6$:Mo^{4+}/Er^{3+}, $Cs_2NaInCl_6$:Mo^{4+}/Tm^{3+} and $Cs_2NaInCl_6$:Mo^{4+}/Yb^{3+} measured at 930 nm with different Ln^{3+} doping concentration under 365 nm excitation, respectively; b), e), h) and k) Luminescence decay curves of $Cs_2NaInCl_6$:Mo^{4+}/Ho^{3+}, $Cs_2NaInCl_6$:Mo^{4+}/Er^{3+}, $Cs_2NaInCl_6$:Mo^{4+}/Tm^{3+} and $Cs_2NaInCl_6$:Mo^{4+}/Yb^{3+} measured at different characteristic emission peaks of Ln^{3+} ions with different Ln^{3+} doping concentration under 365 nm excitation, respectively; c), f), i) and l) The ET efficiencies of $Cs_2NaInCl_6$:Mo^{4+}/Ho^{3+}, $Cs_2NaInCl_6$:Mo^{4+}/Er^{3+}, $Cs_2NaInCl_6$:Mo^{4+}/Tm^{3+} and $Cs_2NaInCl_6$:Mo^{4+}/Yb^{3+} from Mo^{4+} to Ln^{3+} in different Ln^{3+} doping concentration, respectively.

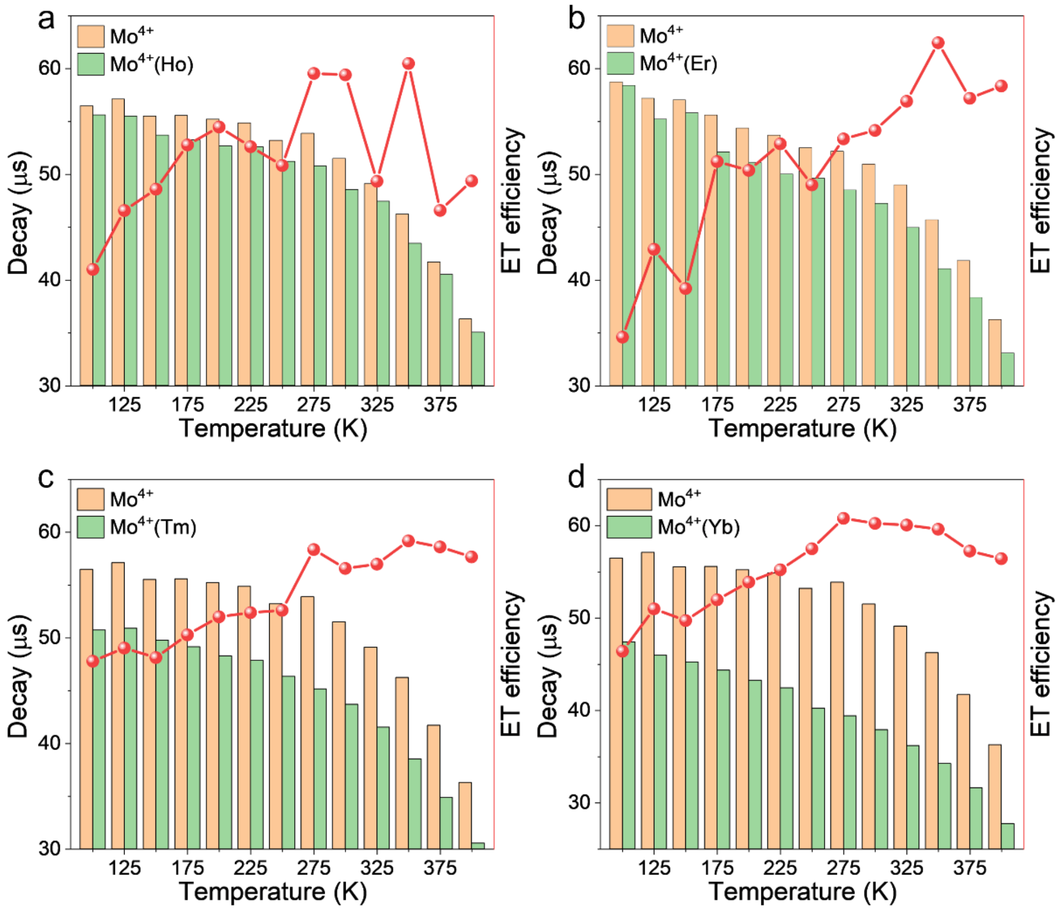

Fig. S30 Temperature-dependent Mo^{4+} lifetimes in $Cs_2NaInCl_6:Mo^{4+}$ and $Cs_2NaInCl_6:Mo^{4+}/Ln^{3+}$, and the corresponding $Mo^{4+}\rightarrow Ln^{3+}$ (Ln = Ho, Er, Tm and Yb) energy transfer efficiency, respectively.

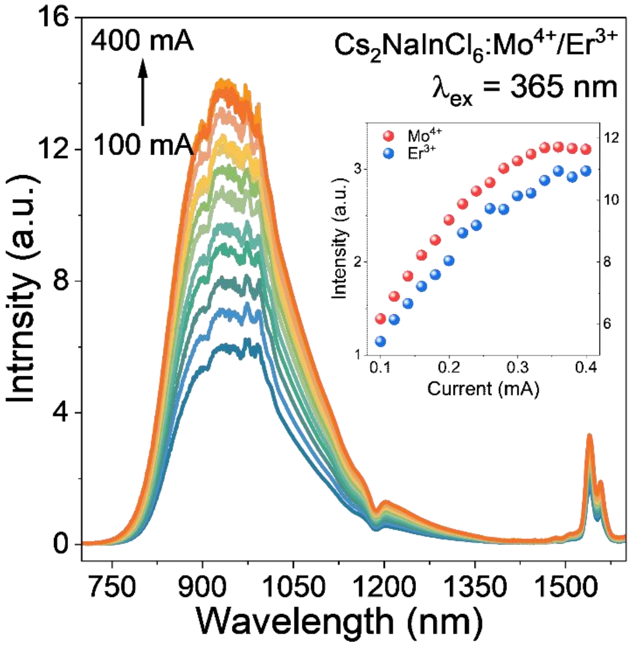

Fig. S31 Driven current dependent emission spectra and luminescence intensity of the as-fabricated NIR pc-LED with 365 nm chip.

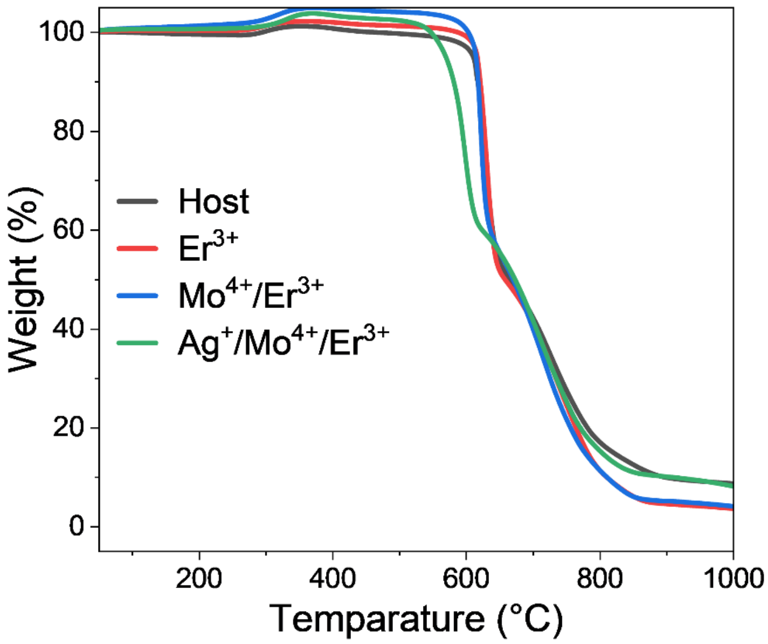

Fig. S32 Thermogravimetric analysis data of host, Er^{3+}-, Mo^{4+}/Er^{3+}- and $Ag^{+}/Mo^{4+}/Er^{3+}$-doped $Cs_2NaInCl_6$, measured under inert atmosphere.

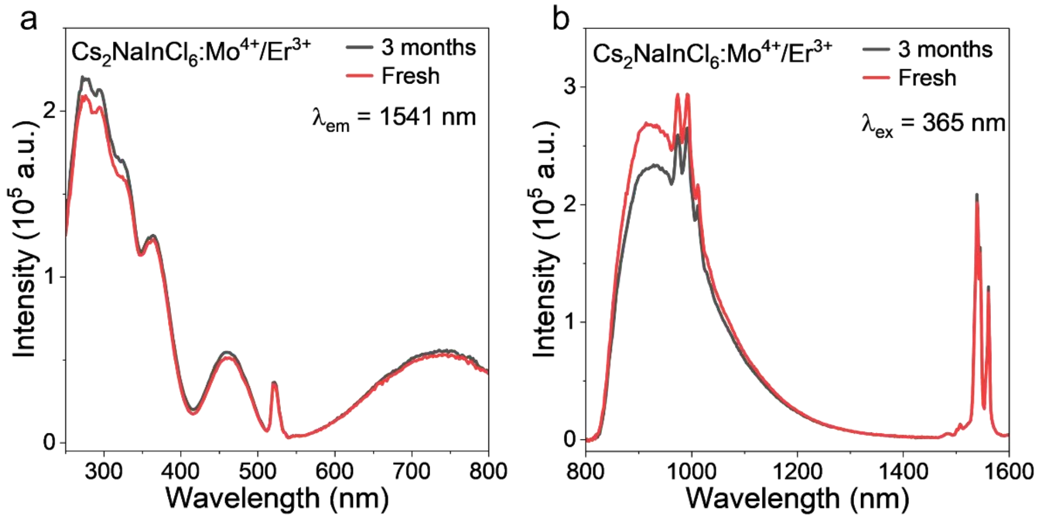

Fig. S33 Comparison of spectral stability of samples stored for three months.

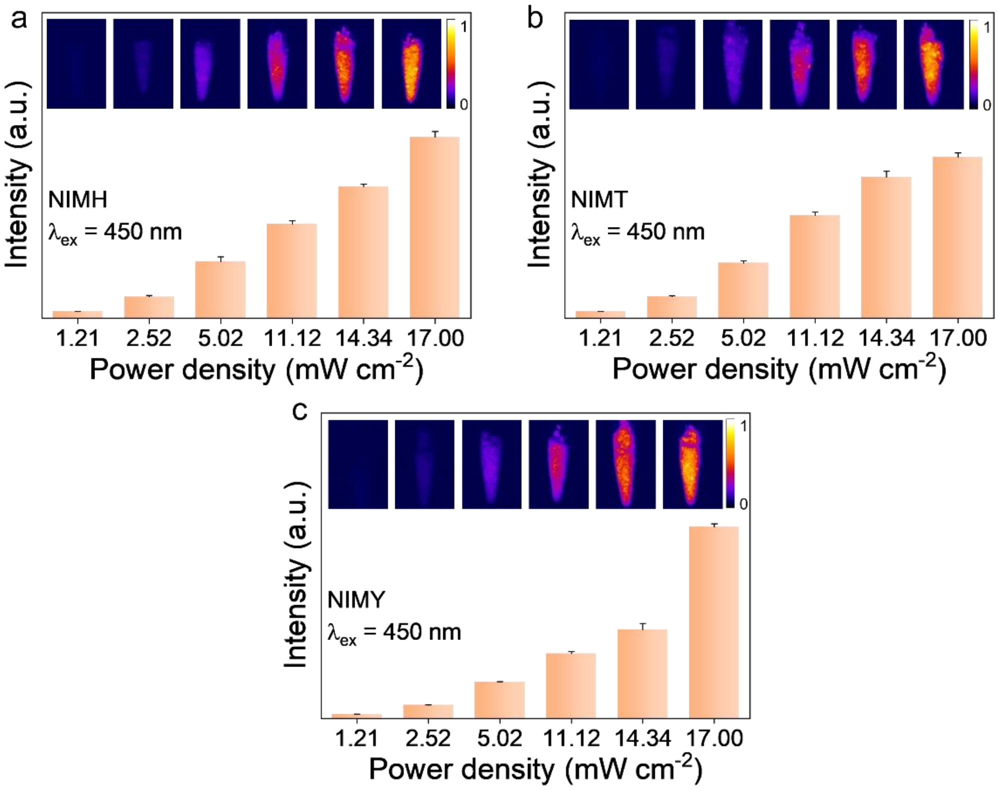

Fig. S34 The histogram of NIR luminescence intensity excited at different power densities. The insets are NIR luminescence images of $Cs_2NaInCl_6$:Mo^{4+}/Ho^{3+}, $Cs_2NaInCl_6$:Mo^{4+}/Tm^{3+} and $Cs_2NaInCl_6$:Mo^{4+}/Yb^{3+} excited with different power densities, respectively.

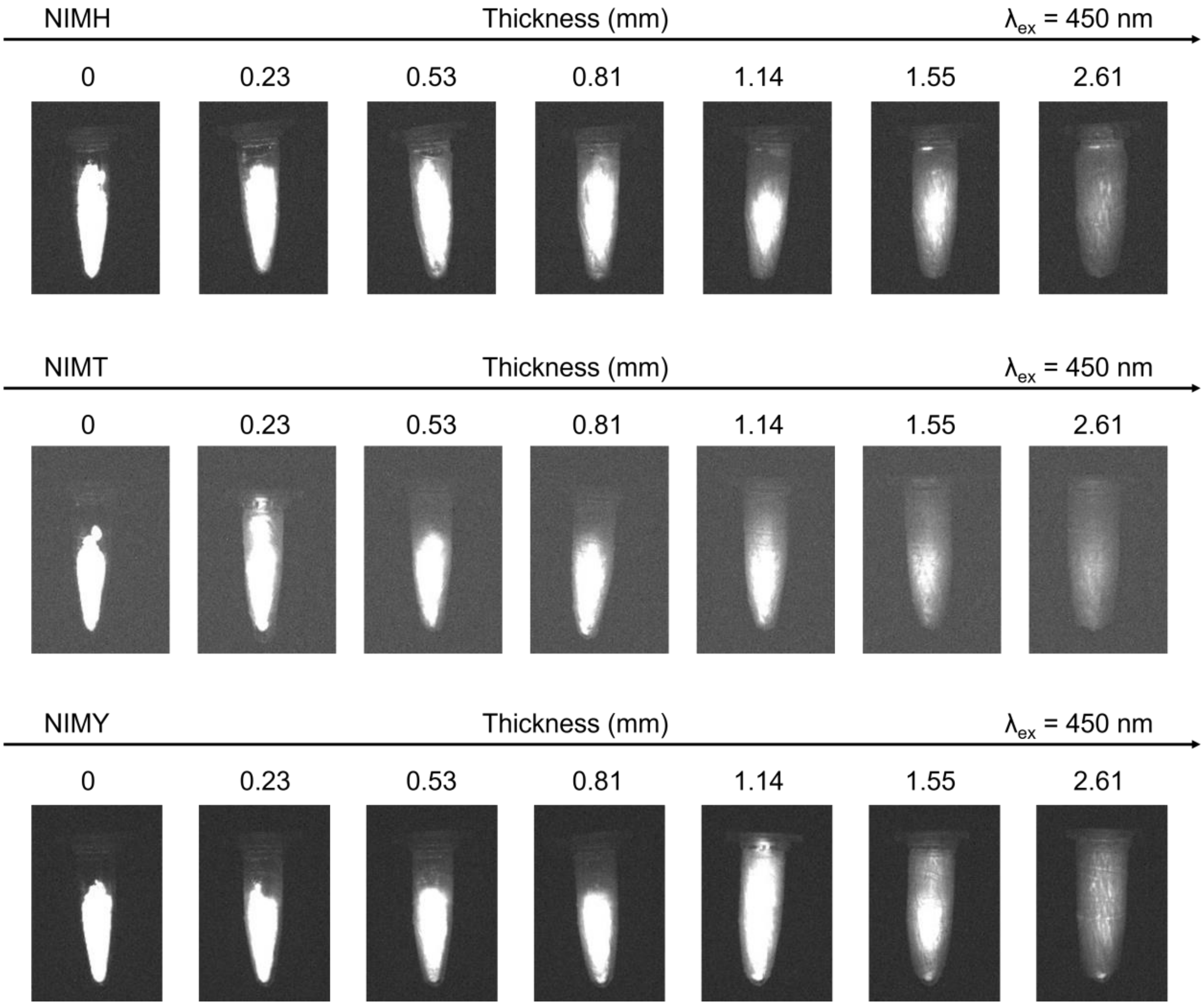

Fig. S35 The NIR luminescence images of $Cs_2NaInCl_6$:Mo^{4+}/Ho^{3+}, $Cs_2NaInCl_6$:Mo^{4+}/Tm^{3+} and $Cs_2NaInCl_6$:Mo^{4+}/Yb^{3+} through seal films of different thicknesses under the excitation of 450 nm, respectively.

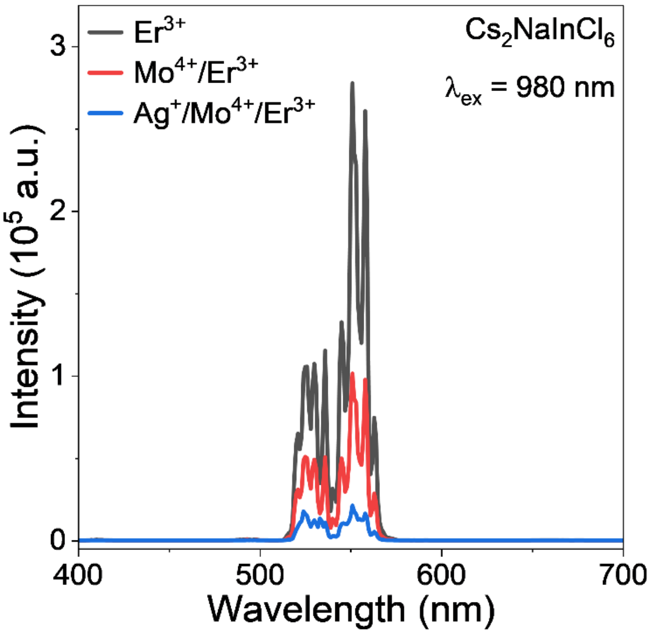

Fig. S36 Up-conversion PL spectra of three Er^{3+}-doped samples.

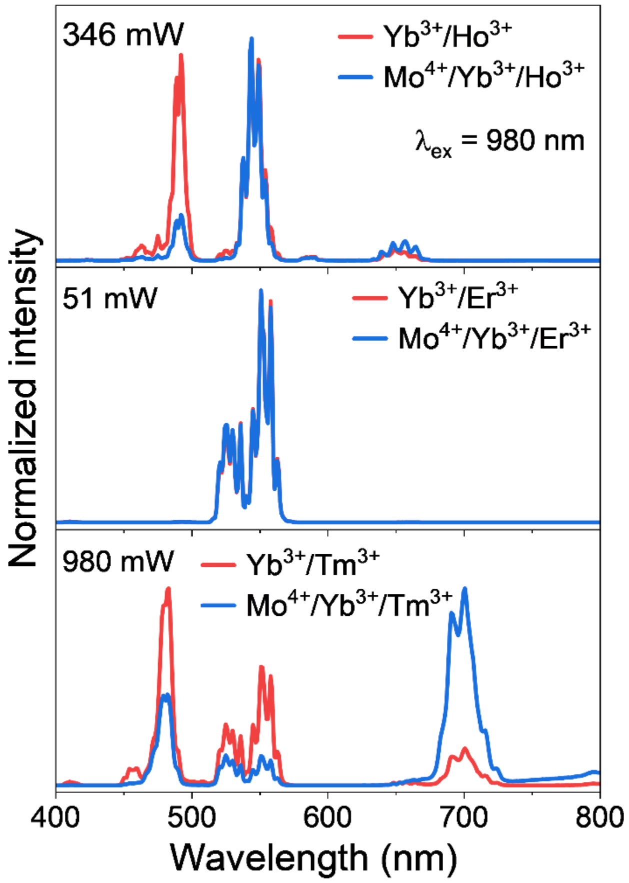

Fig. S37 Up-conversion PL spectra of Yb^{3+}/Ln^{3+}-doped samples.

References

1. Jin, S. *et al.* Boosting STE and Nd^{3+} NIR Luminescence in $Cs_2AgInCl_6$ Double Perovskite via Na^+/Bi^{3+}-Induced Local Structure Engineering. *Advanced Functional Materials* **33**, 2304577 (2023).
2. Nie, J. *et al.* Modulation of the near-infrared-I and -II luminescence of thulium-incorporated lead-free double perovskites. *Inorganic Chemistry Frontiers* **11**, 6960-6969 (2024).
3. Chen, D. *et al.* Simultaneous enhancement of near infrared luminescence and stability of $Cs_2AgInCl_6:Cr^{3+}$ double perovskite single crystals enabled by a Yb^{3+} dopant. *Inorganic Chemistry Frontiers* **9**, 4695-4704 (2022).
4. Pei, Y. *et al.* Boosting Near-Infrared Luminescence of Lanthanide in $Cs_2AgBiCl_6$ Double Perovskites via Breakdown of the Local Site Symmetry. *Angewandte Chemie International Edition* **61**, e202205276 (2022).
5. Zhao, C., Gao, Y., Song, T., Wang, J. & Qiu, J. An Er^{3+}-Doped $Cs_2NaScCl_6$ Lead-Free Double Perovskite with Efficient Broadband Visible to Near-Infrared Emission and Multimodal Upconversion Luminescence. *The Journal of Physical Chemistry Letters* **14**, 9011-9018 (2023).
6. Han, S. *et al.* Unveiling Local Electronic Structure of Lanthanide-Doped $Cs_2NaInCl_6$ Double Perovskites for Realizing Efficient Near-Infrared Luminescence. *Advanced Science* **9**, 2203735 (2022).
7. Yang, X. *et al.* Five-level anti-counterfeiting based on versatile luminescence of tri-doped double perovskites. *Nano Research* **17**, 9971-9979 (2024).
8. Gan, W. *et al.* Broad-Band Sensitization in Cr^{3+}-Er^{3+} Co-Doped $Cs_2AgInCl_6$ Double Perovskites with 1.5 μm Near-Infrared Emission. *Chemistry of Materials* **35**, 5291-5299 (2023).
9. Wang, H. *et al.* Simultaneously achieving multicolor emission of down-shifting and up-conversion in Yb^{3+},Er^{3+}-codoped $Cs_2NaGdCl_6$ double perovskites. *Advanced Optical Materials* **11**, 2300694 (2023).
10. Dang, P. *et al.* Red-NIR Luminescence in Rare-Earth and Manganese Ions Codoped $Cs_4CdBi_2Cl_{12}$ Vacancy-Ordered Quadruple Perovskites. *Chemistry of Materials* **35**, 1640-1650 (2023).
11. Wang, Y. *et al.* Highly efficient visible and near-infrared luminescence of Sb^{3+},Tm^{3+} co-doped Cs_2NaYCl_6 lead-free double perovskite and light emitting diodes. *Journal of Alloys and Compounds* **947**, 169602 (2023).
12. Sun, J. *et al.* Efficient Near-Infrared Luminescence in Lanthanide-Doped Vacancy-Ordered Double Perovskite Cs_2ZrCl_6 Phosphors via Te^{4+} Sensitization. *Angewandte Chemie International Edition* **61**, e202201993 (2022).
13. Yun, X. *et al.* Enabling efficient near-infrared emission in lead-free double perovskite via a codoping strategy. *Inorganic Chemistry Frontiers* **11**, 6146-6155 (2024).
14. Gan, W. *et al.* Manganese ion-sensitized near-infrared light in $Cs_2NaBi_{1-x}Er_xCl_6$ lead-free double perovskite. *Advanced Optical Materials* **10**, 2102851 (2022).
15. Wang, Y., Hou, H., Bai, Y., Zou, B. & Zeng, R. Thermal enhanced energy transfer and high thermal sensitivity of Sb^{3+} doped Cs_2KYbCl_6 rare earth double perovskites. *Laser & Photonics Reviews* **18**, 2400337 (2024).
16. Lian, B. *et al.* Multimode luminescence with temperature and energy level synergistic dependence in rare earth halide DPs for advanced multifunctional applications. *Small* **20**, 2401093 (2024).
17. Huang, W. *et al.* Efficient Near-Infrared Luminescence with Near-Unity Photoluminescence

Quantum Yield in Erbium-Doped Double Perovskites Cs_2NaYCl_6 under Green Light Excitation. *Chemistry of Materials* **36**, 2483-2494 (2024).

18. Zhu, F., Gao, Y. & Qiu, J. High performance NIR-I to NIR-II emission of a Cr^{3+}-doped $Cs_2NaLuCl_6$ phosphor with an IQE and EQE of up to 92.9% and 60.75%. *Inorganic Chemistry Frontiers* **11**, 7098-7109 (2024).
19. Yang, S., Wei, Y., Wang, Q., Sun, Z. & Li, A.-H. Tm^{3+}-doped $Cs_2Ag_{0.6}Na_{0.4}In_{0.9}Bi_{0.1}Cl_6$ microcrystals for thermometry. *Journal of Alloys and Compounds* **968**, 172212 (2023).
20. Guo, X. *et al.* Tailoring the Emission Properties of $Cs_2NaLuCl_6$ for Anti-Counterfeiting, X-Ray Scintillation, and Night Vision Applications. *Advanced Optical Materials* **13**, 2402845 (2025).
